# Strategic optimization of conditions for the solubilization of GST-tagged amphipathic helix-containing ciliary proteins overexpressed as inclusion bodies in *E. coli*

**DOI:** 10.1186/s12934-022-01979-y

**Published:** 2022-12-12

**Authors:** Amruta A. Shendge, Jacinta S. D’Souza

**Affiliations:** School of Biological Sciences, UM-DAE Centre for Excellence in Basic Sciences, Kalina campus, Santacruz (E), Mumbai, 400098 India

**Keywords:** Recombinant proteins, Inclusion body protein, Amphipathic helices, Solubility, *Escherichia coli*

## Abstract

Expression of affinity-tagged recombinant proteins for crystallography, protein–protein interaction, antibody generation, therapeutic applications, etc. mandates the generation of high-yield soluble proteins. Although recent developments suggest the use of yeast, insect, and mammalian cell lines as protein expression platforms, *Escherichia coli* is still the most popular, due mainly to its ease of growth, feasibility in genetic manipulation and economy. However, some proteins have a spontaneous tendency to form inclusion bodies (IBs) when over-expressed in bacterial expression systems such as *E. coli*, thus posing a challenge in purification and yield. At times, small peptides undergo degradation during protein production and hence using suitable tags could circumvent the problem. Although several independent techniques have been used to solubilize IBs, these cannot always be applied in a generic sense. Although tagging a GST moiety is known to enhance the solubility of fusion proteins in *E. coli*, resulting in yields of 10–50 mg/L of the culture, the inherent nature of the protein sequence at times could lead to the formation of IBs. We have been working on a Myc Binding Protein-1 orthologue, viz. Flagellar Associated Protein 174 (FAP174) from the axoneme of *Chlamydomonas reinhardtii* that binds to an A-Kinase Anchoring Protein 240 (AKAP240) which has been annotated as Flagellar Associated Protein 65 (FAP65). Using an in-silico approach, we have identified two amphipathic helices on FAP65 (*Cr*FAP65AH1 and *Cr*FAP65AH2) that are predicted to bind to FAP174. To test this prediction, we have cloned the GST-tagged peptides, and overexpressed them in *E. coli* that have resulted in insoluble IBs. The yields of these over-expressed recombinant proteins dropped considerably due to IB formation, indicating aggregation. An integrated approach has been used to solubilize four highly hydrophobic polypeptides, viz. two amphipathic helices and the respective proline variants of FAP65. For solubilizing these polypeptides, variables such as non-denaturing detergents (IGEPAL CA-630), changing the ionic strength of the cell lysis and solubilization buffer, addition of BugBuster^®^, diluting the cell lysate and sonication were introduced. Our statistically viable results yielded highly soluble and functional polypeptides, indiscreet secondary structures, and a yield of ~ 20 mg/L of the *E. coli* culture. Our combinatorial strategy using chemical and physical methods to solubilize IBs could prove useful for hydrophobic peptides and proteins with amphipathic helices.

**Supplementary Information:**

The online version contains supplementary material available at 10.1186/s12934-022-01979-y.

## Introduction

Proteins are thought of as the ‘workhorses’ of the cell, playing important roles in biological activities like metabolism, DNA replication, transcription, translation, DNA repair, gene regulation, and signalling. They also make up the structure of the cell. Genetic modification methods that produce physiologically active recombinant proteins are typically used to fully comprehend each of their distinct activities. An ever-expanding field of recombinant DNA technology has developed to achieve this goal, allowing for the design or modification and production of high-yield proteins. With the aid of these techniques, scientists have created valuable proteins that can be used to study the physicochemical, molecular, structure–function, and biological functions of many proteins [1]. Steps for obtaining a recombinant protein include selecting the gene of interest and cloning it in a suitable vector, transforming it into an expression host, and inducing and purifying it to homogeneity. A recent development suggests the use of yeast, mammalian, and insect cell lines over traditional expression systems such as bacteria and fungi for acquiring eukaryotic proteins that require post-translational modifications [2]. In contrast to *E. coli*, mammalian cell lines provide a further advantage by their ability to secrete the protein in the medium making the down-stream process simpler [2]*.* Although being the most popular over-expression platform, *E. coli* poses several drawbacks, it lacks a post-translational modification system, has a distinct codon bias, exhibits RNA-instability, forms IBs, may contain lipopolysaccharides that are associated with the purified protein and may interfere with some of its uses, etc. [2]. Earlier, IB formation was a valuable tool for obtaining active recombinant proteins, however, its extraction and solubilization depend on numerous factors such as the presence of osmolytes, molecular chaperones, stabilizing enzymes and effective post-translational modification machinery leading to biologically active proteins. Despite the development of several tools to predict protein solubility, the tendency of a protein to form IB cannot be accurately determined. These IBs can cause intrinsic toxicity and conformational stress to the cells producing them. Therefore, the formation of IBs can pose an important obstacle during the production of recombinant proteins. Since IBs tend to aggregate, they can eventually reduce the solubility of the target protein [3]. In spite of these disadvantages, *E. coli* remains the most preferred prokaryotic expression system because it has the advantages of genetic manipulation, well-optimized expression, rapid growth, and low cost, thereby making it the most sophisticated prokaryotic expression system [4]. To achieve efficient protein expression, the promoter should allow the recombinant protein to accumulate up to 10–30% of the total cellular protein. As described earlier, the formation of IBs during the induction of the recombinant protein is one of the major disadvantages of using *E. coli* as an over-expression platform. These IB proteins lack their inherent biological activity because they form aggregates and are difficult to purify and refold into their native conformation(s). To improve the expression, solubilization, and purification of recombinant proteins, it is therefore advised to alter the culture conditions by switching the medium or the temperature, co-expressing chaperones, adding large hydrophilic fusion tags as well as adding sucrose, raffinose, glycine, betaine and sorbitol during growth for enhancing the recombinant protein expression, solubilization and purification [5, 6].

Recombinant proteins have a variety of fusion tags attached to their N- or C-termini. These fusion proteins or tags serve as chaperones, assisting their protein folding and solubilization of the target proteins. Another fusion tag with the albumin binding domain, streptococcal protein G (SPG), stabilizes short-lived proteins as a result of the binding of serum albumin, which has a longer half-life [7]. By translocating the fusion protein to various cellular regions with fewer proteases, other tags, including maltose-binding proteins and tiny ubiquitin-related modifier fusion partners can prevent it from being degraded [8, 9]. To stop self-aggregation, these chaperones tend to bind to the hydrophobic portion(s) of the partially folded proteins. An 8 kDa calcium-binding protein extracted from the parasite *Fascicola hepatica* is employed as a tag for the synthesis of soluble proteins in one such fusion system (Fh8). The stability and ability to purify proteins are attributes of the first 11 amino acids at the N-terminus region of the Fh8 tag. As a result, a smaller (1 kDa) H tag was created to match these corresponding to these 11 amino acids. This tag is also well known for its ability to increase solubility [10]. *E. coli* over-expression vectors have fusion tags, the translational initiation site, the 5′ untranslated region (5′ UTR), the antibiotic selection marker, the transcription promoter site, and the origin of replication, just like any other expression vector. For successful protein expression, the promoter must be strong and enable accumulation of the recombinant protein up to 10–30% of the total cellular protein. In the past, tags were big proteins that were resistant to proteolytic degradation, which improved the production and solubility of heterologous proteins. For example, a fusion protein with a 1024 amino acid tag, such as LacZ, might be affinity purified on p-amino-phenyl-β-d-thio-galactosidase (APTG) column and eluted with a high pH borate buffer. Most proteins with this tag, though, are insoluble [11]. Poly histidine tag is the most prevalent and cost-effective affinity tag for generating significant quantities of recombinant proteins from *E. coli*, as compared to certain widely used tags like FLAG tag and Strep-II tag [12]. The majority of commercially available polyhistidine tags can span from 2 to 10 histidines, which can form coordination bonds with metal ions such as Co^2+^, Ni^2+^, Cu^2+^, Zn^2+^, Ca^2+^, etc. More than 80% of pure protein is obtained by overexpressing proteins with this affinity tag in *E. coli* than compared to mammalian and insect cell lines wherein there are more chances of proteins having stretches of histidine residues thereby purifying nonspecific proteins in the affinity chromatography step [12]. Glutathione S-transferase (GST), a 26 kDa protein, isolated primarily from the parasitic helminth *Schistosoma japonicum*, is one of the oldest tags and has a high affinity for glutathione [13]. The GST fusion tag has been widely utilized to enhance protein solubility and protect the protein from proteolytic degradation while achieving native protein folding. It is typically positioned at either the C- or N-terminus of the protein of interest [13]. Efficient initiation of translation is demonstrated in a wide range of overexpression platforms such as *E. coli* [14, 15], yeast [16, 17], plants [18, 19] and mammalian cells when full-length or truncated proteins are expressed as fusion tags [20, 21]. The simplicity with which GST-fusion proteins can be purified to homogeneity in a one-step affinity chromatography using glutathione immobilized to a matrix (or support) and glutathione itself serving as the eluant further lends support to the choice of GST. Denaturing electrophoresis, followed by western blotting and immunoprobing with commercially available anti-GST antibodies, can also be used to quickly evaluate purified GST-tagged proteins [13]. Another disadvantage of the GST tag is its large size (218 a.a.) and the propensity to dimerize in a solution that may affect the properties of the fusion protein [13]. Small and large-sized tags, including poly-Histidine (19 a.a.), Myc epitope (11 a.a.), maltose binding protein (396 a.a.), Glutathione S-transferase (GST) (211 a.a.), small ubiquitin-like modifier (SUMO) (100 a.a.), galactose binding protein (GBP) (509 a.a.), etc., have all been reported to increase protein expression and solubility [11]. Calculating the pI of the recombinant protein is also necessary because it can be used to tune the buffer pH for ion exchange chromatography [22].

As was previously described, one of the biggest downsides of using *E. coli* as an over-expression platform is the formation of IBs during the induction of recombinant proteins. The formation of IB protein is not an abnormal phenomenon; rather, it is one of the manifestations of protein misfolding which may result from partially folded polypeptides that fail to achieve their native stable conformation or aggregation of native protein which has less solubility [23]. It is clear that a number of circumstances, such as a high concentration of overexpressed protein, a lack of post-translational mechanisms, reducing conditions in the cytoplasm, a lack of interaction with enzymes or chaperones necessary for protein folding, etc. [23] contribute in the formation of IB. Hofmeister discovered more than ten decades ago that differences in the solubility of proteins occur with a variety of different salts. Lindwall et al. optimized the buffer composition in an effort to extract and solubilize non-aggregated proteins. Arranging the ions from the least to the most chaotropic ones, he observed that ammonium sulfate can both stabilize the proteins in the folded state as well as extract them in the solution [24]. The high salt concentration of sodium sulfate, sodium acetate, and magnesium sulfate can stabilize protein or reduce its solubility or salts such as potassium thiocyanate, magnesium chloride and calcium chloride can denature the protein or increase its solubility whereas salts such as sodium chloride and potassium chloride may or may not act as a stabilizer [25, 26]. Any given salt’s ability to stabilize or destabilize a protein depends on the ratio of the exposed polar or nonpolar groups on its surface [26]. On the other hand, the propensity of divalent salts as agents of salting-in or salting-out is produced by a delicate balance between the preferential hydration or exclusion exhibited by the surface free energy of water and the binding of the cation to the protein [27]. During protein purification, the use of high concentrations of chaotropic agents such as urea (> 4 M) and guanidine hydrochloride (> 1.5 M) results in protein denaturation and leads to aggregation, thus creating misfolded protein during the refolding process [22]. Proteins with more hydrophobic amino acids are more likely to form aggregates. Several chromatographic techniques are typically used to separate soluble proteins with > 50% yield. However, utilizing solubilization buffers with a range of pH and ionic strength (0.01–1 M NaCl concentration) as well as detergents such as 1–5% of Triton X-100 and 10 mM CHAPS, IB proteins are recovered up to low to a decent amount of yield (5–20%) [22]. These IBs form electron refractile particles that take cylindrical to ovoid shapes to fit in the *E. coli* cells and are dispersed throughout the cytoplasm and periplasm [28]. However, the addition of glycerol, sucrose and other polyhydric alcohols increases the stability of the protein by helping in protein folding [29]. Although IB protein purification is considered undesirable, they have some major advantages too. They are resistant to proteolytic degradation by cellular proteases and can be easily separated based on their density with a high expression level as compared to other cellular proteins.

**Fig. 1:**
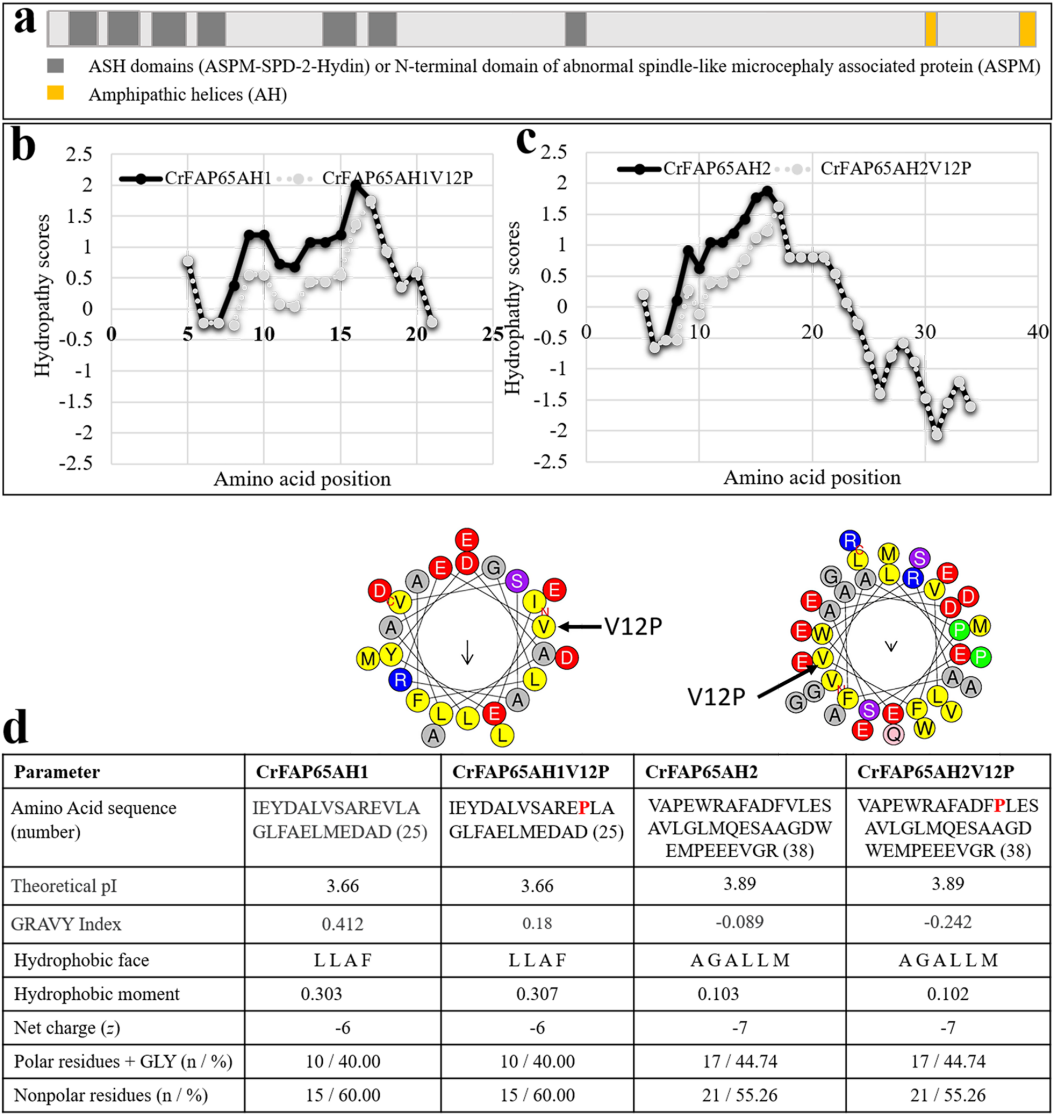
**a** Cartoon representation of *Cr*FAP65 primary sequence with the ASH domains (grey filled boxes) and amphipathic helices (yellow filled boxes) and the hydropathy plots (**b**, **c**) derived for all the four polypeptides without the GST tag. This was carried out using an *in-silico* tool (https://web.expasy.org/protscale/ and https://web.expasy.org/protparam/). Note the presence of 7 ASH domains and 2 amphipathic helices. **d** In silico parameters and helical wheel projection using HeliQuest (https://heliquest.ipmc.cnrs.fr/)

A Myc Binding Protein (FAP174) which is an RII-like protein and an A-Kinase Anchoring Protein (FAP65) from the axoneme of the green chlorophyte, *Chlamydomonas reinhardtii* are the two ciliary proteins that are the subject of the current study. We are trying to map the domains of interaction between these two ciliary proteins as a part of an ongoing research program. A-kinase anchoring proteins use their amphipathic helices to bind with high affinity to the regulatory subunit of Protein kinase A. The Dimerization and Docking (D/D) domain which is the characteristic feature of these regulatory subunits (RII) has been classically used to detect cellular AKAPs [30]. The 93 a.a. long stretch of FAP174 has been shown to interact directly with an A-Kinase Anchoring Protein, viz. AKAP240 [31]. The protein has now been annotated as FAP65 [32]. This protein (FAP65) from *C. reinhardtii* has also been predicted to have seven abnormal spindle-like microcephaly-associated or ASPM-SPD-2-Hydin (ASH) domains (Fig. 1a). These domains are a part of the Pap-D-like superfamily [33]. As an AKAP interactor, it is hypothesized that FAP174 harbours the D/D domain that binds to the amphipathic helices predicted to be present on FAP65. Hence, using in silico strategies and the Heliquest tool, two amphipathic helices were identified [33], cloned, and over-expressed as GST fusion polypeptides in the hope that these highly hydrophobic sequences would not form IBs. However, *Cr*FAP65AH1, *Cr*FAP65AH2 and their proline variants (*Cr*FAP65AH1V12P and *Cr*FAP65AH2V12P) all produced IBs when overexpressed in *E. coli*. As a result, a systematic strategy was adopted to solubilize the GST-tagged proteins and bypass the refolding step, thereby shortening the time between the growth of cells until dialysis of the purified protein. The purified proteins would serve as a source in the domain mapping of the amphipathic helices of FAP65 with FAP174.

## Materials and methods

All chemicals and media components were procured from Sigma or Millipore-Merck or SRL, India. The bacterial strains *E. coli* DH5α and *E. coli* BL21 DE3 used for this study were procured from Genei (Bangalore, India) and Stratagene (C607003, Thermofisher Scientific, USA).

### Bacterial strains, plasmids, and transformation of E. coli cells

The target genes *Cr*FAP65AH1, *Cr*FAP65AH2 and their variants were gene synthesized by GenScript (https://www.genscript.com/) in the pGEX-4T-1 vector. *E. coli* DH5α strain was transformed with the expression vector pGEX 4T-1 with individual commercially synthesized (Genscript USA Inc.) genes of *Cr*FAP65AH1, *Cr*FAP65AH1V12P, *Cr*FAP65AH2 and *Cr*FAP65AH2V12P giving rise to Glutathione S-transferase (GST) tagged fusion polypeptides (for details see plasmid construct in Additional file 1: Fig. S1). Plasmids were individually extracted from the transformed colonies and were subsequently used to transform competent *E. coli* BL21 cells. *E coli* transformation was carried out using the chemical transformation method. Briefly, 100 μL of overnight *E. coli* broth culture was inoculated in 10 ml fresh LB broth. Aliquots of culture were collected at an interval of 1 h and the optical densities were determined at 600 nm. When the optical densities reached 0.4, the culture was chilled for 10–15 min following which they were transferred to pre-chilled microfuge tubes and centrifuged at 4950*g*/10 min at 4 °C. The supernatant was discarded carefully and the cells were resuspended in 3 ml of chilled (80 mM CaCl_2_ and 50 mM MnCl_2_) buffer and kept on ice for 30 min. The cells were again centrifuged at 2200*g*/10 min at 4 °C. The supernatant was discarded carefully and the cells were resuspended in 200 µL of chilled CaCl2 (80 mM) and kept on ice for 2 h. These cells were transformed with 1.0 μL of the DNA (100 ng/μL) by the heat shock method (42 °C for 90 s). The colonies obtained after selection on LB medium (5 g/L yeast extract, 5 g/L NaCl, 10 g/L tryptone, 20 g/L agar) containing ampicillin were patched onto agarified LB medium containing 100 mg/L ampicillin.

### Protein sequence analysis

Analysis of protein sequences such as *Cr*FAP65AH1, *Cr*FAP65AH2 and their respective variants *Cr*FAP65AH1V12P and *Cr*FAP65AH2V12P was carried out using an in-silico approach. To determine the hydrophobicity of the sequence, a PROT Scale tool based on Kyte and Doolittle hydrophobic scale was used (https://web.expasy.org/protscale/). Hydrophobicity indices were determined as the Grand average of Hydropathicity (GRAVY) values of hydrophobic regions using the ProtParam tool (https://web.expasy.org/protparam/) [34].

### Cell growth and induction of protein expression

One single colony of each of the four transformed clones was transferred to a 10 ml LB liquid medium containing 100 mg/L of ampicillin and grown overnight at 37 ℃. The next day, 2 ml of each of the cultures was added to 100 ml LB liquid medium containing 100 mg/L of ampicillin and allowed to grow at 37 ℃ until the optical density at 600 nm (OD_600_) reaches between 0.4 and 0.6. Following this, 1 mM iso-propyl β-D-1-thiogalactopyranoside (IPTG) was added. Cells were harvested at 1, 3, and 6 h after induction, and an uninduced sample was also harvested before the addition of IPTG.

### Observation of inclusion bodies in E. coli using transmission electron microscope (TEM)

*Escherichia coli* cells harvested after 6 h of IPTG were centrifuged at 2000*g* for 5 min. and resuspended in 1X PBS (0.137 M Sodium chloride, 0.0027 M Potassium chloride, 0.01 M Sodium phosphate dibasic, 0.0018 M Potassium phosphate monobasic) just before the samples were analyzed. ~ 2 µL of the sample, corresponding to ~ 200 cells were placed on the TEM copper grid and analyzed using the Tecnai G^2^ instrument.

### Buffers used for the various steps in the solubility of IBs

LSB-1: 50 mM Tris–Cl pH 7.4, 1 mM EDTA, 10 mM β-mercaptoethanol, 1% Triton-X 100, 300 mM NaCl, 1 mM Phenylmethanesulfonyl fluoride (PMSF) (Sigma, Catalogue-P7626) and 500 μL BugBuster^®^ Protein Extraction Reagent (70,584 Merck-Millipore).

LSB-2: 10 mM Tris–Cl, pH 7.4, 1 mM EDTA, 10 mM β-mercaptoethanol, and 1 mM PMSF.

LSB-3: 10 mM Tris pH 7.4, 1 mM EDTA, 10 mM β-mercaptoethanol, 150 mM NaCl, and 1 mM PMSF.

### Formation of inclusion bodies and solubility testing

Following induction with IPTG, *E. coli* cells were centrifuged at 2000*g*/10 min., supernatant discarded and cells washed 1X with fresh Luria Bertani liquid medium. For the solubility test, cell pellet from 250 ml (⁓2 × 10^12^ cells) culture was harvested and 1250 μL of a lysis and solubilization buffer containing 50 mM Tris–Cl pH 7.4, 1 mM EDTA, 10 mM β-mercaptoethanol, 1% Triton-X 100, 300 mM NaCl, 1 mM PMSF and 500 μL BugBuster^®^ Protein Extraction Reagent was added, maintaining the ratio of cells to buffer as ~1.6 × 10^12^ cells/ml of buffer. This lysis and solubilization buffer is called LSB-1. The homogenate was incubated for 1 h/RT and sonicated for 10 cycles of 30 s each with 60 s interval at 4 ℃ followed by centrifugation at 20,000*g*/30 min. The pellet and supernatant were re-suspended in an SDS-PAGE sample buffer and all samples were electrophoresed on 12% denaturing gel at a constant voltage.

### Solubilization strategy

For solubilization, changes were made to the LSB-1 mentioned earlier. The components that were retained were 10 mM Tris–Cl, pH 7.4, 1 mM EDTA, 10 mM β-mercaptoethanol, and 1 mM PMSF. This lysis and solubilization buffer was called LSB-2. To this LSB-2, different NaCl concentrations (75, 150 and 300 mM), IGEPAL CA-630 (1%) and increased buffer volume (1250, 1875 and 3750 μL, respectively giving rise to ratios of ~ 1.6 × 10^12^, ~ 1.06 × 10^12^, ~ 0.53 × 10^12^ cells/ml of buffer) for a 250 ml culture pellet was used along with BugBuster^®^ Protein Extraction Reagent and Sonication conditions were tested.

### Purification of the GST-fusion polypeptides

In a 2 ml sterile Eppendorf tube, 1000 μL (bed volume 800 μL) of Glutathione S-sepharose beads were centrifuged at 375*g* at 4 °C/1 min. Glutathione Sepharose™ 4B affinity chromatography resin was procured from Cytiva (Product code 17-075-605). The supernatant was discarded and the beads were washed thrice with LSB-2 containing 150 mM NaCl which is termed LSB-3 (10 mM Tris pH 7.4, 1 mM EDTA, 10 mM β-mercaptoethanol, 150 mM NaCl, and 1 mM PMSF) with or without IGEPAL CA-630 followed by incubation at 4 °C/1 h. After 1 h of equilibration, the beads were added to the supernatant and were incubated on a cell mixer at 4 °C/1 h for binding. Meanwhile, the column was rinsed with MilliQ and LSB-3. Following 1 h of binding, the beads along with the supernatant were added to the rinsed column (Econo-Pac^®^ Disposable Chromatography Columns, Bio-Rad Catalogue-732-1010) and allowed to settle down for 2 min. The flow-through containing the unbound proteins was collected. Lysis and solubilization buffer-3 was used as the wash buffer so that the non-specifically bound contaminants are washed out. The GST-fusion protein was then eluted with an elution buffer [10 mM reduced Glutathione (Sigma Catalogue no. G4251) in 150 mM NaCl, 10 mM Tris pH 7.4, 1 mM EDTA, 10 mM β-mercaptoethanol and 1 mM PMSF]. The eluted protein was then dialyzed against 10 mM Tris and 150 mM NaCl (pH 7.4). For dialysis, sacks (Sigma Catalogue no. D6191) of 12 kDa cut-off were used. After dialysis, the protein concentration was estimated using Bradford’s reagent with BSA as a standard followed by aliquoting and storing at − 80 °C until further use.

### Circular dichroism of proteins

All the purified proteins were individually dialyzed at 4 °C, checked for the GST tag using an anti-GST antibody, and CD spectra were measured (JASCO-CD Spectropolarimeter J-815 Serial no. A029961168) in a cuvette with 1 mm path length, 195–260 nm with a bandwidth of 1 nm, and scanning speed of 100 nm/min. The protein samples (0.1 mg/ml) were dialyzed with a buffer containing 10 mM Tris and 50 mM NaCl, pH 7.4. The chamber was continuously flushed with N_2_ gas. The resultant spectral values are expressed as Molar ellipticity. The BeStSel tool was used to understand the detailed secondary structure of the protein by analyzing the CD spectra (http://bestsel.elte.hu/index.php). This tool uses CD spectra of protein which is nothing but differential absorption between the right and left circularly polarized light. It also provides an estimate of the secondary structure of elements of the protein such as helix, β sheet, turn and disordered.

### Pull-down assay

In this assay, a GST fused polypeptide (bait) immobilized on a glutathione-conjugated resin is used to determine its interacting partners (prey) from unpurified samples or unknown protein samples. This rapid, in vitro method, helps not only to purify the recombinant protein but also to determine its binding partners [35]. For this purpose, 2.5 mg each of purified *Cr*FAP65AH1, *Cr*FAP65AH2 and their respective variants were incubated for 2 h/4 °C with Glutathione-Sepharose beads (~ 50 µl bed volume) and the flow-through allowed to drain. This was followed by washes with LSB-3 until no protein was present in the washes. The beads were then incubated overnight at 4 ℃ with purified FAP174 followed by collecting the flow-through and washes. GST was used as a positive control and FAP174 was used as a negative control, to eliminate any non-specifically bound proteins to the beads during pull-down. Finally, an aliquot of the beads was then mixed with 2X SDS-PAGE sample buffer, heated, and electrophoresed using SDS-PAGE.

### Dot blot overlay

To demonstrate the biological activity of the purified recombinant proteins, an overlay assay was performed wherein increasing concentrations of purified 6XHis-tagged *Cr*FAP174 protein was spotted directly onto a nitrocellulose membrane. The dot blot was then allowed to air dry followed by 1 h of blocking with 3% Bovine Serum Albumin (BSA, SRL Catalogue no. 9048-46-8) in TBST [10 mM Tris, 150 mM NaCl and 0.05% Tween20**®** (Sigma Catalogue no. P9416)]. Proteins such as *Cr*FAP65AH1, *Cr*FAP65AH2, *Cr*FAP65AH1V12P, *Cr*FAP65AH2V12P and GST were affinity purified and individually overlaid on the blots at a concentration of 5 μg/ml in TBST containing 1% BSA), overnight (16 h at 4 °C). Following which the blots were washed thrice with TBST. They were then incubated with shaking conditions for 1 h at RT with primary antibody (Monoclonal Anti-GST tag antibody produced in rabbit Merck, Catalogue no. A7340). After three 10 min. washes in TBST, a secondary antibody (Goat anti-Rabbit IgG Antibody-HRP conjugate, Sigma Catalogue no. 12-348) was added and incubated at RT for 1 h. The bands or spots were detected using Clarity™ Western ECL Substrate (Biorad Catalogue no. 1705060). Controls included blots that were not overlaid with the bait but incubated with primary and secondary antibodies or secondary antibodies alone.

### MALDI-TOF mass spectrometry

One μL of 10 mg/ml α-Cyano-4-hydroxycinnamic acid (HCCA, Sigma Catalogue no. C8982) made in TA30 solvent (30:70 [v/v] acetonitrile: 0.1% TFA in water) was mixed with 1 μL of 1 μM each of *Cr*FAP65AH1, *Cr*FAP65AH2, *Cr*FAP65AH1V12P, *Cr*FAP65AH2V12P. 0.5 μL of this mixture was deposited on the MALDI-TOF target plate and allowed to dry. Once dried, the MALDI-TOF plate was inserted into the mass spectrometer followed by laser ablation of the samples under vacuum. Spectra generated for the samples allowed the identification of the molecular sizes of the respective proteins.

### ImageJ analysis

The gels that are marked for ImageJ analysis were converted into a high-quality jpg image and dragged to open onto the ImageJ window. To adjust the histogram of the image, the subtract background is used from the ‘Process’ menu thereby reducing the background for further processing. Although quantification is not considered very accurate, a relative percentage is always reliable. For quantifying the bands of interest, the rectangular selection tool is used to select the band of interest usually in the first lane (UI). After this selection, the corresponding induced protein (band of interest) in the second lane (I) is further selected followed by the third and subsequent lanes. When all the lanes are selected, the Analyze/Gels/Plot lanes menu is used to obtain semi-quantitative values. The wand tool is then used to select the area under the curve, this leads to an absolute value for each band. For calculating the relative values, the induced band is considered as 100 and the other values are plotted as a percentage relative to the ‘induced' value.

### Statistical analysis (ANOVA and Tuckey)

The mean and variance for each of the total soluble protein concentrations in the supernatants and yields/L of *E. coli* culture were calculated. To investigate whether the differences in the concentration of total soluble protein and the yields were statistically significant for the different combinatorial strategies of purification, One-way ANOVA was performed with Tuckey’s method that uses studentized range distribution [36]. The significance level was set at α = 0.05. The results so obtained for each parameter are shown in the Tables for each graph. It may be noted that each condition has been repeated three times (3 biological replicates) and each time three technical replicates have been performed.

## Results

### In silico insight into the amphipathic helices of CrFAP65

The FAP65 sequence was identified from Phytozome (https://phytozome-next.jgi.doe.gov/) with a gene ID of Cre07.g354551 and was shown to harbour two such helices henceforth termed *Cr*FAP65AH1 (25 a.a., 2.74 kDa) and *Cr*FAP65AH2 (38 a.a., 4.22 kDa) (Fig. 1a, d). Two proline variants at the 12th position for each of these sequences were also synthesized. Such variants were designed since proline ‘kinks’ in the peptide thereby inhibiting the interaction. These helices and their variants when purified as recombinant proteins would serve as a useful resource in protein interaction studies. Knowing that these are amphipathic in nature and that overexpression in *E. coli* might pose a problem, we set out to investigate in silico parameters to gain further insights. Various physicochemical parameters such as the total charge (− 6 for *Cr*FAP65AH1 and − 7 for *Cr*FAP65AH2), the pI (3.66 for *Cr*FAP65AH1 and 3.89 for *Cr*FAP65AH2), the number of polar (40% for *Cr*FAP65AH1 and 44.74% for *Cr*FAP65AH2) and non-polar residues (60% for *Cr*FAP65AH1 and 55.26% for *Cr*FAP65AH2) were estimated by HeliQuest (https://heliquest.ipmc.cnrs.fr/). The Grand average of hydropathicity (GRAVY) index score was also determined. It may be noted that the GRAVY index score is a measure of the average hydrophobicity and hydrophilicity of proteins measured by the Kyte-Doolittle Formula [37]. The GRAVY index and other in silico parameters for each protein sequence were also identified using the ProtParam tool. The GRAVY index measures the ratio of the sum of hydropathy values of all a.a. to the length of the protein. A hydrophobicity score is an arbitrary unit in which, below zero indicates the likelihood of the protein of interest to be globular (i.e. more hydrophilic), while scores above zero indicate the proteins be membranous (i.e. more hydrophobic). When applied to *Cr*FAP65 amphipathic helices, the GRAVY index for *Cr*FAP65AH1 and *Cr*FAP65AH2 were 0.412 and − 0.089, respectively indicating that the former was more hydrophobic than the latter. To further ascertain the hydropathicity for these sequences, hydropathy values of each a.a. residues were plotted for *Cr*FAP65AH1, *Cr*FAP65AH2 and their variants (*Cr*FAP65AH1V12P and *Cr*FAP65AH2V12P; Fig. 1b, c). These plots indicate that the *Cr*FAP65AH1 and its variant are largely hydrophobic in nature as compared to *Cr*FAP65AH2 and its variant. The number of hydrophobic amino acids in *Cr*FAP65AH1 are 11 in number, while those in *Cr*FAP65AH2 are 23, thus making both the peptide sequences highly hydrophobic [ProtParam (https://web.expasy.org/protparam/)]. Our analysis showed two prominent hydrophobic peaks in all four proteins. Since we are dealing with amphipathic helices, we set about using Heliquest to make the helical wheel and determine the hydrophobic moment (μH, Fig. 1d). The latter is the mean vector sum of the side-chain hydrophobicities of a given helix with N number of a.a. residues. It is 0.303 and 0.307 for *Cr*FAP65AH1 and its variant, respectively. The hydrophobic moment drops to 0.103 and 0.107 for *Cr*FAP65AH2 and its variant, respectively (Fig. 1d). This indicated that *Cr*FAP65AH1 and its variant were more amphipathic in nature as compared to *Cr*FAP65AH2 and its variant. Given all these in silico inputs, it was decided to choose a tag such as GST (pGEX-4T-1 vector) which could in principle solubilize the polypeptides and refrain it m forming IBs when overexpressed in a host such as *E. coli*.

### Induction and inclusion body formation of the CrFAP65 clones

**Fig. 2:**
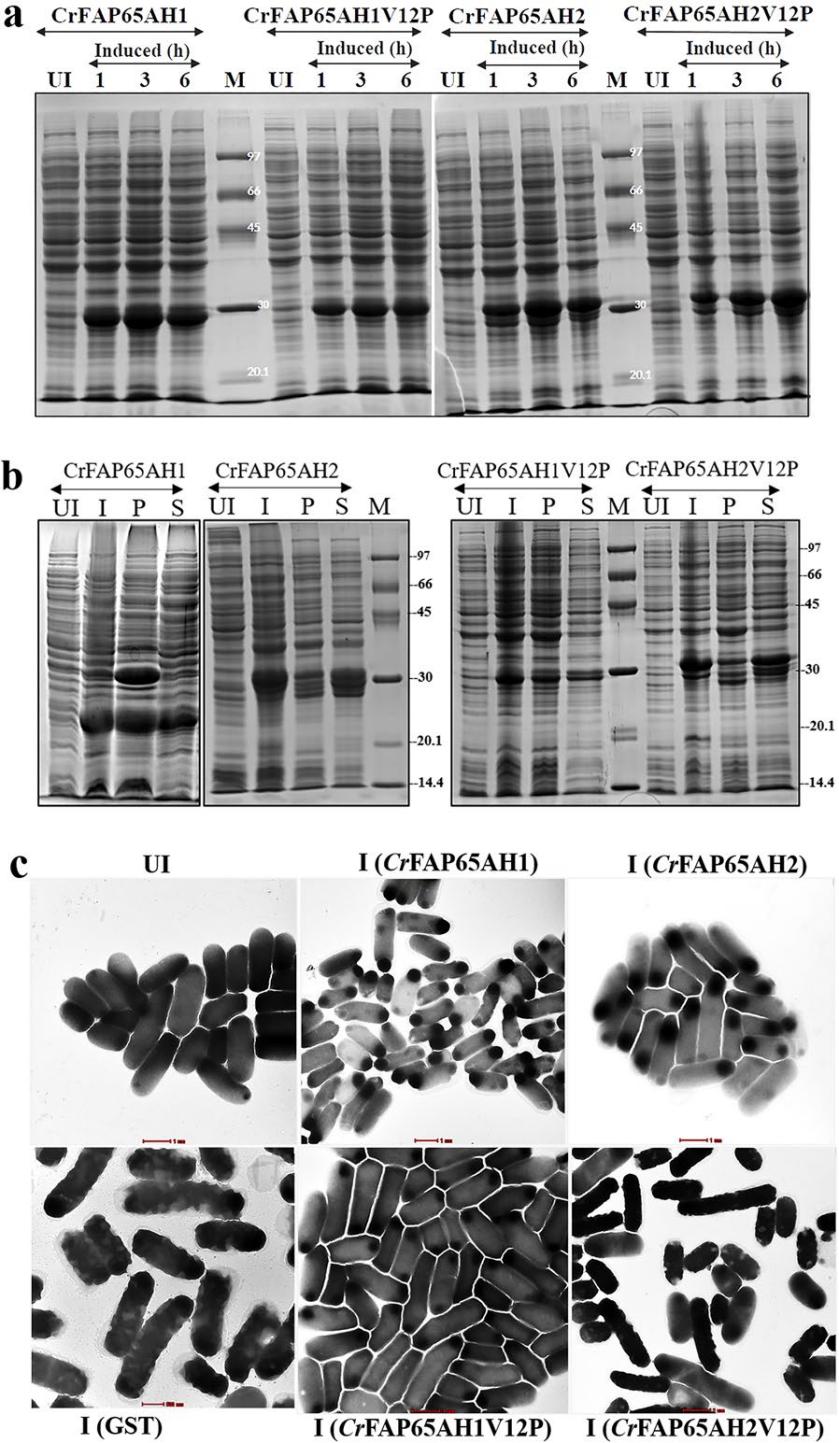
Kinetics of induction of the recombinant proteins (**a**) and formation of insoluble fusion polypeptides (**b**). **a** Uninduced (UI) *E. coli* cell lysate and kinetics of induction of the recombinant proteins at 1, 3 and 6 h. **b** Uninduced (UI), induced (I) *E. coli* cells and protein fraction of pellet (P) and supernatant (S) after lysis followed by centrifugation. Both pellets and supernatants were then electrophoresed using 12% SDS-PAGE. The molecular weights of *Cr*FAP65AH1 and *Cr*FAP65AH1V12P is 29.4 kDa and that of *Cr*FAP65AH2 and *Cr*FAP65AH2V12P is 30.64 kDa. **c** Transmission electron microscopy images of *E. coli* BL21DE3: Uninduced *E. coli* BL21 DE3 cells, Induced *Cr*FAP65AH1, Induced *Cr*FAP65AH2, Induced GST, Induced *Cr*FAP65AH1V12P, Induced *Cr*FAP65AH2V12P. The brightness and contrast features for the gels have been adjusted between 12 and 16%

Once the sequences of the transformed clones were verified for their authenticity, cells were grown and induced with IPTG (1 mM) and the cell pellet was checked for induction on a denaturing gel (Fig. 2a). All four independent clones of *Cr*FAP65AH1, *Cr*FAP65AH2, *Cr*FAP65AH1V12P and *Cr*FAP65AH2V12P showed abundant overexpression at 1, 3 and 6 h of induction. The molecular weights with the GST tag (26 kDa) on the denaturing gel for *Cr*FAP65AH1 and *Cr*FAP65AH1V12P (both, 29.4 kDa), and *Cr*FAP65AH2 and *Cr*FAP65AH2V12P (both, 30.64 kDa) were as expected. The next obvious step was to check for the solubility of the overexpressed recombinant fusion proteins (Fig. 2b). For the amphipathic helices of FAP65, the conventional laboratory procedure of lysis using sonication in LSB-1 (containing 300 mM NaCl and BugBuster^®^) was used. The latter treatment did solubilize the recombinant proteins to some extent (Fig. 2b). However, when the individual supernatants for the fusion proteins were used for purification, the yields obtained were ~ 4–6 mg/L of *E. coli* culture (data not shown). This yield was found to be substantially lower than those reported for most GST-tagged fusion proteins [21]. It was observed that most of the protein remained in the pellet which means most of the protein did not solubilize due to IB formation. Therefore, sub-cloning these genes in a vector (pET28a) having a smaller tag (6X His) was attempted in parallel. However, no visible induction was obtained (data not shown). Hence, it was decided to pursue using pGEX-4T-1 for expressing these genes in *E. coli*. To confirm that the protein forms IBs in *E. coli* cells, TEM analysis was performed of uninduced *E. coli* BL21-DE3 cells, *E. coli* cells inducing GST, *Cr*FAP65AH1, *Cr*FAP65AH1V12P, *Cr*FAP65AH2 and *Cr*FAP65AH2V12P gene. The electron-dense particles (IBs) were observed at the extreme corners in the induced *E. coli* BL21-DE3 cells expressing *Cr*FAP65AH1, *Cr*FAP65AH1V12P, *Cr*FAP65AH2 and *Cr*FAP65AH2V12P protein. In the *E. coli* cells inducing GST, these electron-dense particles were seen throughout the cytoplasm whereas no such electron-dense particles were seen in any uninduced *E. coli* cells (Fig. 2c). Induction was also performed at a lower temperature i.e. at 20 °C using 0.1 mM IPTG. However, as observed under a transmission electron microscope, IBs were still evident (Additional file 2: Fig. S2). To improve their respective yields, a systematic strategy involving the ‘design of experiments approach' for solubilization was developed by first changing the ionic strength of the LSB-1 that contains 300 mM NaCl. Decreasing the NaCl concentration will give rise to a salting-in effect thereby solubilizing the protein further. These improvisations were first tested on *Cr*FAP65AH1. Using different reagents for each solubilization experiment, the target outcomes for each were monitored after incubating the induced cell pellets with the solubilization buffer containing the reagent followed by centrifugation and electrophoresis on a denaturing SDS-PAGE gel. An outcome was monitored by semi-quantitative ImageJ analysis of the band corresponding to the GST-fusion polypeptide (mentioned as the band of interest) from the treatments of the various supernatants.

### Effect of increasing the ionic strength and diluting the cell lysate in the presence and absence of BugBuster^®^ without sonication

**Fig. 3:**
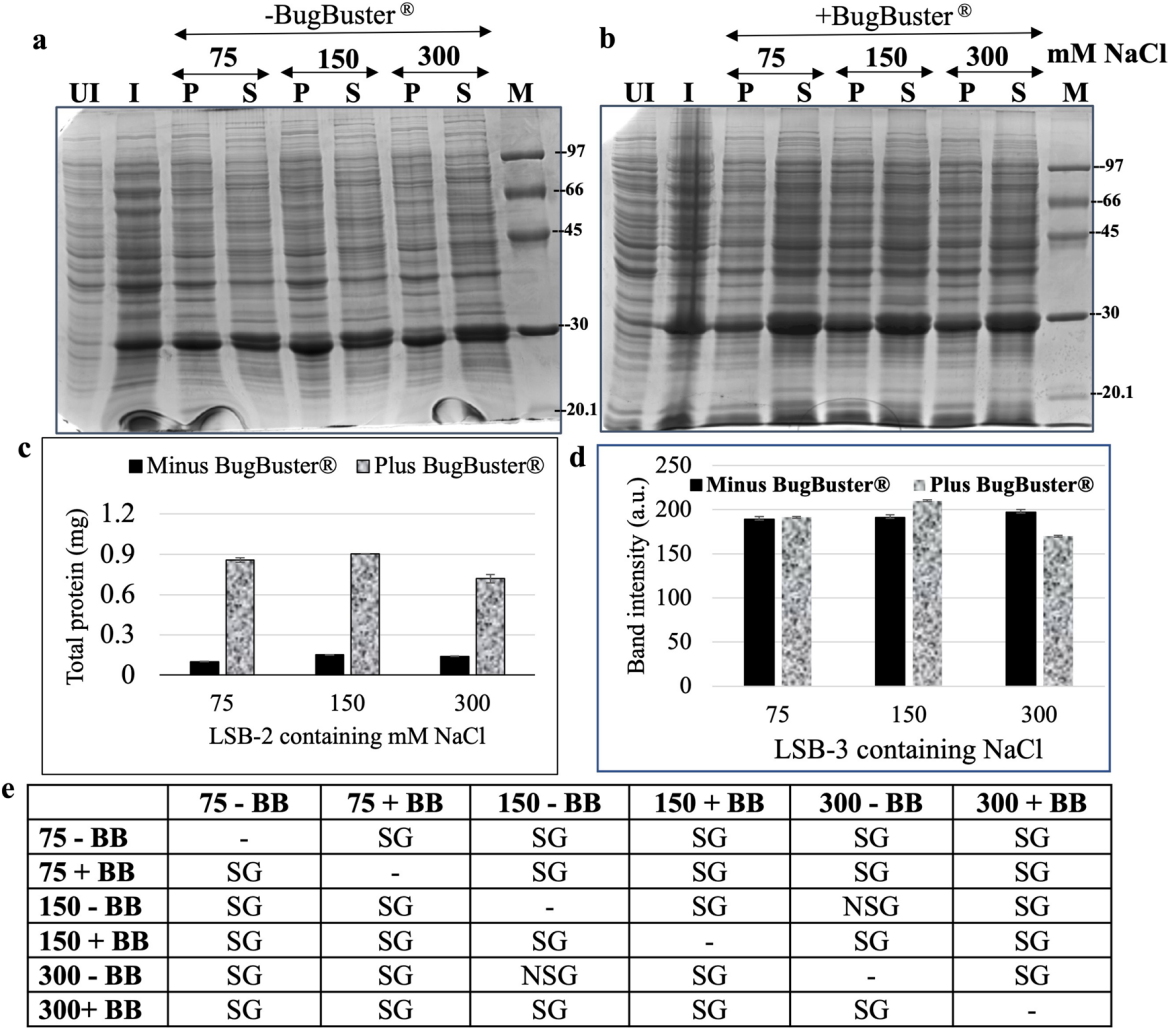
The effect of increasing NaCl (75, 150 and 300 mM) concentration in the absence and presence of BugBuster^®^ (BB) keeping the buffer volume constant at 1250 μL. **a**, **b** Induced pellets after 6 h of induction were either incubated with LSB-2 with or without BugBuster^®^ and increasing concentration of NaCl. Post-centrifugation, the pellets and supernatants were subjected to SDS-PAGE. **c** The total soluble protein concentration of the supernatants, both with or without BugBuster^®^ was estimated using Bradford’s assay and plotted. Note the 6 to 9-fold increase in the total soluble protein concentration in the supernatants when BugBuster^®^ is used. **d** The induced fusion protein band (the band of interest) of the SDS-PAGE gels were analyzed using the ImageJ tool, both with and without BugBuster^®^. **e** Statistical analysis of the total soluble protein in the supernatants from treatments with and without BugBuster^®^. BB, NSG and SG, respectively stand for BugBuster^®^, significant and non-significant values as analyzed using one-way ANOVA and Tuckey method. Statistically significant difference between the group means F(5,12) = 2140.42, p = 2.89E^−17^, F_critical value_ = 3.10, wherein F statistic (between,df_within_) = F ratio, p-value, F-critical value. The molecular weights of *Cr*FAP65AH1 and *Cr*FAP65AH1V12P is 29.4 kDa and that of *Cr*FAP65AH2 and *Cr*FAP65AH2V12P is 30.64 kDa. For both the gel images, the brightness and contrast have been adjusted to 20%

Induced cells were next used in triplicates and re-suspended independently in LSB-2 containing 75, 150 or 300 mM NaCl keeping all the other components (10 mM Tris, 1 mM EDTA, 10 mM β-mercaptoethanol and 1 mM PMSF) intact, followed by incubation with BugBuster^®^ and processed without any sonication. The pellets after solubilization and supernatants for each treatment were electrophoresed on a denaturing gel (Fig. 3a, b). Although the salting-in effect was observed in samples treated with LSB-2 containing 75 and 150 mM NaCl, the total soluble protein content of the supernatants revealed that samples treated with LSB-2 containing 150 mM NaCl extracted maximum total soluble protein content in the supernatants (Fig. 3c), and those treated with 300 mM showed an inhibiting and therefore a salting-out effect. The overall solubilization for all proteins significantly increased (~ eightfold) in the presence of BugBuster^®^ (Fig. 3c) indicating that the proprietary formulation provided by the manufacturer worked to disrupt *E. coli* cells and released several proteins as it contains non-ionic detergents. At this stage, it was unclear if all the *E. coli* cells were disrupted and whether the retained *Cr*FAP65AH1 in the pellet was because of compromised disruption of cells or because the protein was not fully solubilized. Semi-quantitative ImageJ analysis of the fusion polypeptide band indicated that the LSB-2 containing 150 mM NaCl with BugBuster^®^ increased the solubilization albeit not very significantly (Fig. 3d). Since it was decided to avoid most of the harsh chaotropic reagents, the next step was to increase the volume of the solubilization buffer. We observed that there was a statistically significant difference between the group means F(5,12) = 2140.42, p = 2.89E^−17^, F_critical value_ = 3.10 (Fig. 3c; Total soluble protein values), wherein F statistic (between,df_within_) = F ratio, p-value, F-critical value.

**Fig. 4:**
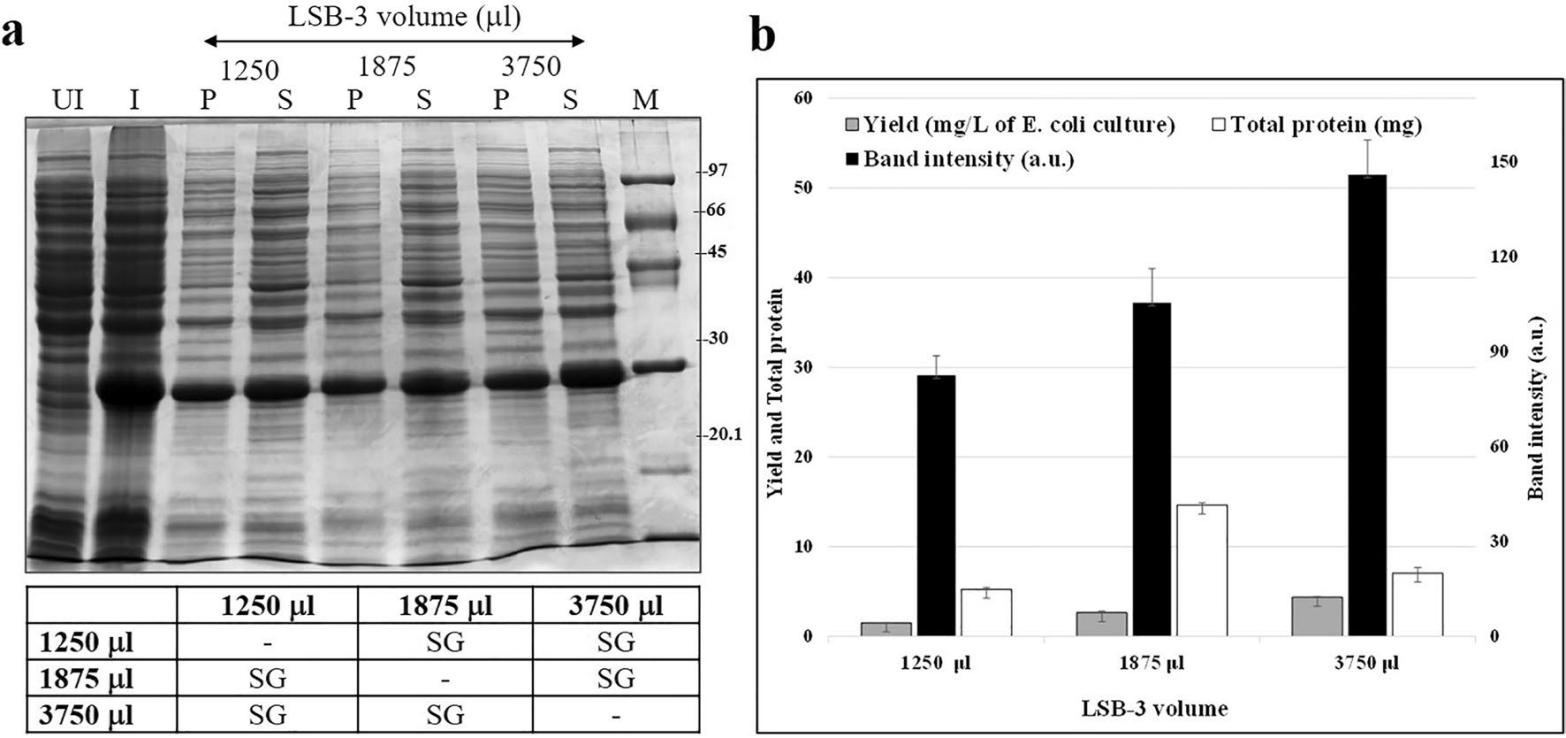
Solubilization of *Cr*FAP65AH1-GST tagged fusion protein with LSB-3 containing 150 mM NaCl and BugBuster^®^ with increasing the volume of the buffer. **a** Induced *E. coli* cells after treatment with increasing buffer volume were processed for electrophoresis using 12% SDS-PAGE. **b** The graph indicates the band intensity of the supernatants for the induced fusion protein from the SDS-PAGE gel of **a**, total soluble protein in the supernatant in mg and pure protein yields post-purification using affinity chromatography. **c** Statistical analysis of the total soluble protein in the supernatants and yield for the increasing volume of LSB-3. NSG and SG, respectively stand for significant and non-significant values as analyzed using one-way ANOVA and Tuckey method. Statistically significant difference between the group means F(2,6) = 380.59, p = 4.78E^−07^, F_critical value_ = 5.14, wherein F statistic (df_between_,df_within_) = F ratio, p-value, F-critical value. Since the band intensities were analyzed using the ImageJ tool, these are relative amounts and hence were not considered suitable for statistical analysis. The molecular weights of *Cr*FAP65AH1 and *Cr*FAP65AH1V12P is 29.4 kDa and that of *Cr*FAP65AH2 and *Cr*FAP65AH2V12P is 30.64 kDa. For the gel image, the brightness and contrast have been adjusted to 20%

In the presence of 150 mM NaCl in LSB-2, the volume of the buffer was increased from 1250 to 1875 and 3750 μL for a cell density of ~ 2 × 10^12^ cells (250 ml culture pellet), (Fig. 4a) avoiding sonication at this point. Close to twofold solubilization (increase) was observed with an increasing volume of the buffer, maximum solubilization was seen when the buffer volume was raised to 3750 μL (Fig. 4b). At this stage, the effect of NaCl and increased buffer volume was monitored by electrophoresis of the pellet and supernatant post the respective treatments. The total protein in the supernatants of respective treatments and the intensity of the solubilized/insolubilized bands across gels was calculated. Nevertheless, to ascertain the success of this experimental design, the diluted cell lysates were also monitored for the yield of pure protein post-affinity purification (Fig. 4b, grey histogram). We observed that there was a statistically significant difference between the group means F(2,6) = 380.59, p = 4.78E^−07^, F_critical value_ = 5.14 (Fig. 4b; Yields, grey bars; Fig. 4c), wherein F statistic (df_between_,df_within_) = F ratio, p-value, F-critical value. The effect of sonication was further determined in the presence and absence of a mild detergent, IGEPAL CA-630. In addition, sonication would also be carried out in the presence and absence (see below) of BugBuster^®^ to determine if the latter could be used as a substitute for sonication.

### Effect of sonication in the presence and absence of BugBuster^®^ and another variable, IGEPAL CA-630

**Fig. 5:**
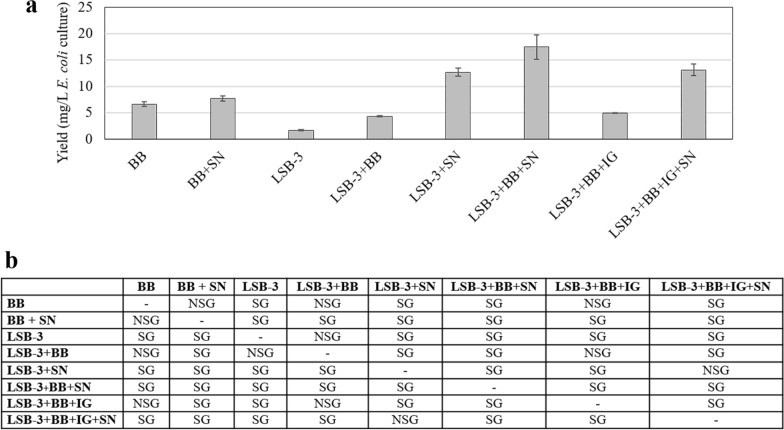
Yield of *Cr*FAP65AH1 GST-fusion protein after affinity purification. **a** Various combination of strategies was followed for the lysis of cells as follows: BugBuster^®^ [BB]; BugBuster^®^ followed by Sonication [BB + SN]; LSB-2 containing 150 mM NaCl, called as LBS-3 [LSB-3]; LSB-3 with BugBuster^®^ [LSB-3 + BB]; LSB-3 followed by Sonication [LSB-3 + SN]; LSB-3 with BugBuster^®^ followed by Sonication [LSB-3 + BB + SN]; LSB-3 with BugBuster^®^ and IGEPAL CA-630 [LSB-3 + BB + IG]; LSB-3 with Bugbuster^®^ and IGEPAL followed by Sonication [LSB-3 + BB + IG + SN] were tested for their solubilization by individually purifying the fusion recombinant protein using affinity chromatography and then calculating the protein content after purification. The graph indicates the yield of the purified fusion protein in each case. **b** Statistical analysis of the yield for different treatments used in the purification. NSG and SG, respectively stand for significant and non-significant values as analyzed using one-way ANOVA and Tuckey method. Statistically significant difference between the group means F(7,16) = 90.83, p = 1.11E−11, F_critical value_ = 2.65, wherein F statistic (df_between_,df_within_) = F ratio, p-value, F-critical value

**Fig. 6:**
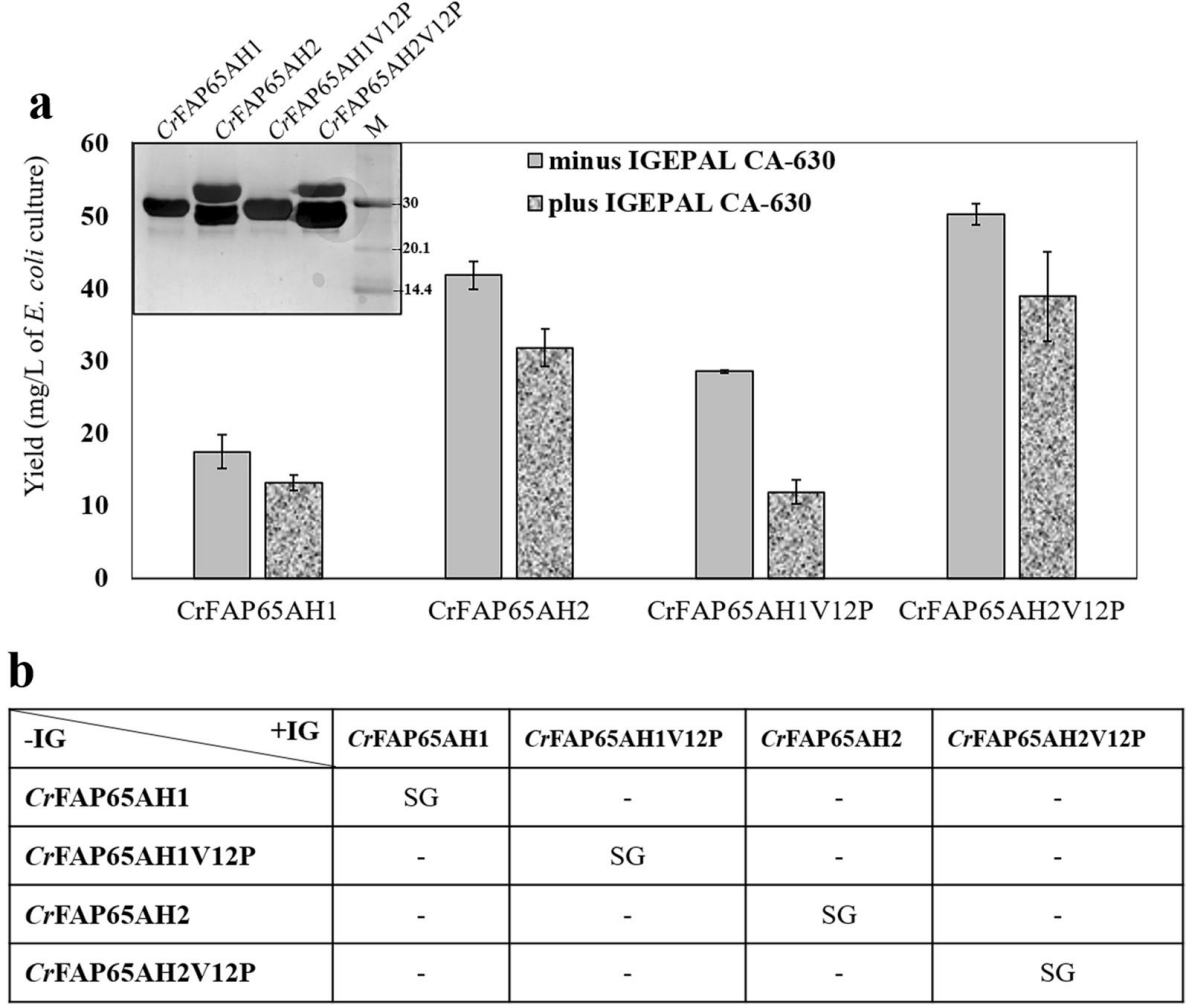
**a** Yields for all four fusion recombinant proteins after affinity purification using the condition of LSB-3 + BugBuster^®^ + SN, 3750 μL volume in the presence and absence of IGEPAL CA-630. Inset is the silver-stained SDS-PAGE gel of the purified fusion proteins. **b** Statistical analysis of the yield for the condition that showed maximum yield with *Cr*FAP65AH1 purification. NS and S, respectively stand for significant and non-significant values as analyzed using one-way ANOVA and Tuckey method. Statistically significant difference between the group means F(1,4) = 8.56, p = 0.0429, F_critical value_ = 7.70 (*Cr*FAP65AH1 ± IGEPAL CA-630); F(1,4) = 302.17, p = 0.000064, F_critical value_ = 7.70 (*Cr*FAP65AH1V12P ± IGEPAL CA-630); F(1,4) = 28.53, p = 0.0059, F_critical value_ = 7.70 (*Cr*FAP65AH2 ± IGEPAL CA-630) and F(1,4) = 9.60, p = 0.036, F_critical value_ = 7.70 (*Cr*FAP65AH2V12P ± IGEPAL CA-630) wherein F statistic (df_between_,df_within_) = F ratio, p-value, F-critical value. The brightness and contrast of the inset gel image has been adjusted to 12%

Since increasing the volume to 3750 μL solubilized > 50% of the protein from the IBs, it was decided to combine BugBuster^®^ with NaCl, a mild detergent (IGEPAL CA-630), and sonication either singly or in combination. The solubilization procedure was followed by centrifugation and affinity purification of the individual recombinant proteins. The yield of the pure protein was calculated after purification for each treatment. Hence, keeping constant the volume of 3750 μL LSB-3, the various parameters that were tested were: BugBuster^®^ (BB), BugBuster^®^ followed by Sonication (BB + SN), LSB-2 containing 150 mM NaCl, henceforth called, Lysis and sonication buffer-3 [LSB-3]; LSB-3 with BugBuster^®^ [LSB-3 + BB]; LSB-3 followed by Sonication [LSB-3 + SN]; LSB-3 with BugBuster^®^ followed by Sonication [LSB-3 + BB + SN]; LSB-3 with BugBuster^®^ and IGEPAL CA-630 [LSB-3 + BB + IG]; LSB-3 with Bugbuster^®^ and IGEPAL followed by Sonication [LSB-3 + BB + IG + SN] (Fig. 5a). When the eluates were pooled and dialyzed, the protein concentration was measured, and yield (per litre of *E. coli* culture) was calculated and plotted. The yield for all these combinations showed a broad range, indicative of the solubilization abilities of BugBuster^®^ alone to sonication in the presence and absence of IGEPAL CA-630 and NaCl. The treatment of LSB-3 increased 20-fold when it contained BugBuster^®^ and was sonicated. The presence of BugBuster^®^ alone with LSB-3 did not make a significant difference. On the other hand, substituting sonication for BugBuster^®^ did make a difference, and a ~ 3.5-fold increase in the yield with sonication was observed (see LSB-3 + BB versus LSB-3 + SN in Fig. 5a). Therefore, BugBuster^®^ could not be used as a substitute for sonication. Nevertheless, BugBuster^®^ alone with and without sonication did not make a significant difference in the yields (see BB versus BB + SN in Fig. 5a). Hence, retaining BugBuster^®^ along with sonication proved useful in solubilizing most of the fusion polypeptide resulting in the highest yield (~ 20 mg/L of *E. coli* culture) among all the conditions tested in this study (Fig. 5a). It may be emphasized that the presence of IGEPAL CA-630 reduced the yield of *Cr*FAP65AH1 as compared to the treatment without IGEPAL CA-630 (see LSB-3 + BB + SN versus LSB-3 + BB + IG + SN in Fig. 5a). To test if this condition was inhibitory for other proteins as well, individual purifications were carried out for other protein pellets in the presence and absence of IGEPAL CA-630 (*Cr*FAP65AH1V12P, *Cr*FAP65AH2 and *Cr*FAP65AH2V12P). The purified protein yields were calculated and a comparison indicated that the best yield was seen for *Cr*FAP65AH2 and its variant which were less hydrophobic than *Cr*FAP65AH1 and its variant (Fig. 6a). To further demonstrate the inhibitory effect of IGEPAL CA-630, all the fusion proteins were solubilized using LSB-3 with BugBuster^®^ and sonicated in the presence of IGEPAL CA-630. When compared with the treatment that did not contain IGEPAL CA-630, inhibition to the tune of 20–30% was observed in all the cases tested (Fig. 6a, dark grey histograms). We observed that there was a statistically significant difference between the group means F(7,16) = 90.83, p = 1.11E-11, F_critical value_ = 2.65 (Fig. 5b), F(1,4) = 8.56, p = 0.0429, F_critical value_ = 7.70 (Fig. 6b) (*Cr*FAP65AH1 ± IGEPAL CA-630), F(1,4) = 302.17, p = 0.000064, F_critical value_ = 7.70 (Fig. 6b) (*Cr*FAP65AH1V12P ± IGEPAL CA-630), F(1,4) = 28.53, p = 0.0059, F_critical value_ = 7.70 (Fig. 6b) (*Cr*FAP65AH2 ± IGEPAL CA-630) and F(1,4) = 9.60, p = 0.036, F_critical value_ = 7.70 (Fig. 6b) (*Cr*FAP65AH2V12P ± IGEPAL CA-630) wherein F statistic (df_between_,df_within_) = F ratio, p-value, F-critical value.

### Quality checks, secondary structures of the purified proteins and bioactivity

**Fig. 7:**
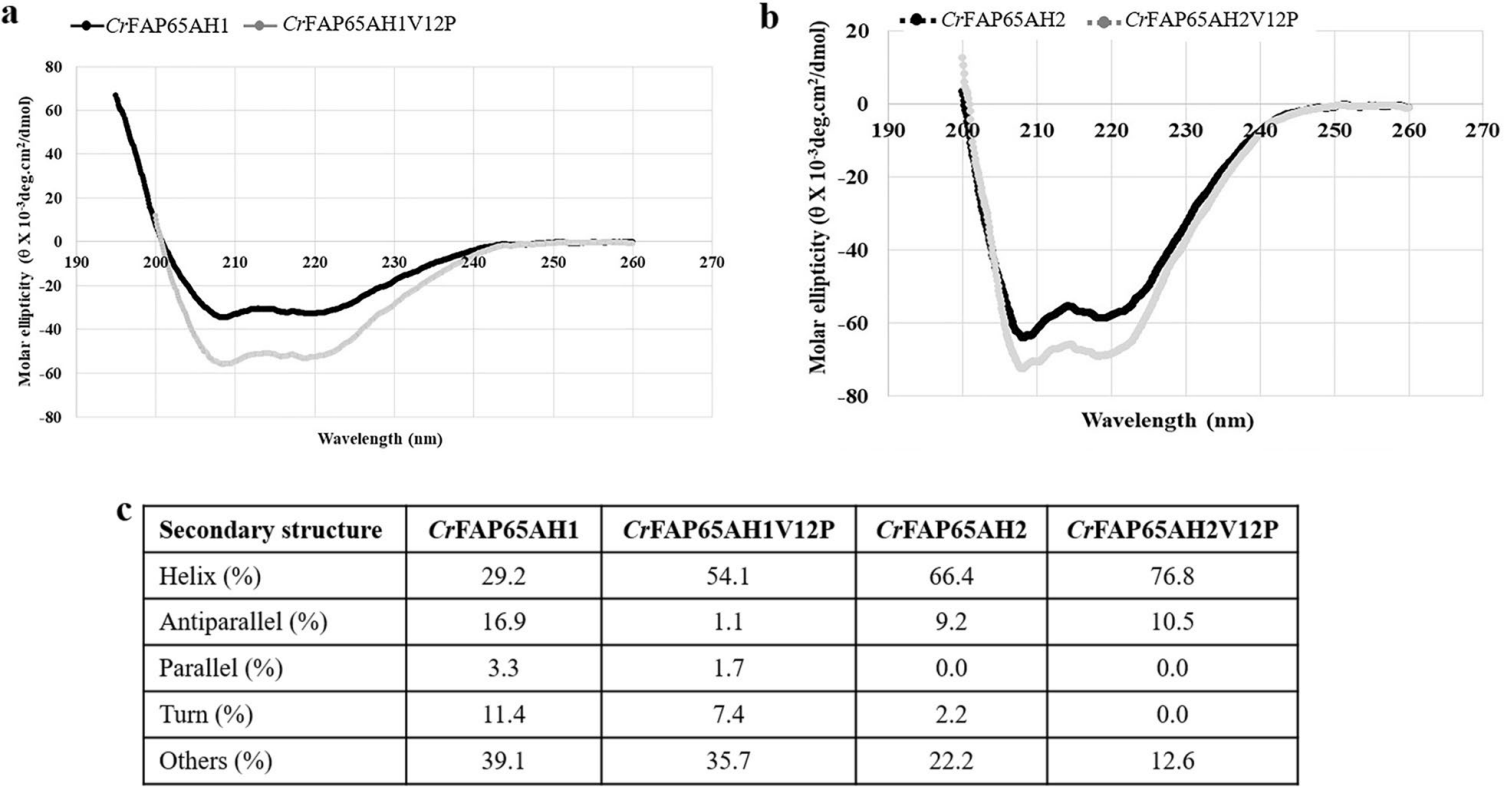
Secondary structure analysis and bioactivity of the fusion proteins using Circular Dichroism. **a**, **b** All four purified GST-fusion proteins were measured for their secondary structures using Circular Dichroism. **c** The alpha-helical content was quantified using the BeStSel tool (see details in the text). Note the increase in alpha helicity with the substitution of the valine with proline in the 12th position for both the amphipathic helices

The proteins thus purified were checked for their molecular weights by electrophoresis on a denaturing gel followed by the sensitive silver staining method (Fig. 6a, inset). Additionally, they were subjected to purity checks and verified for their precise molecular weight (*Mr*) using MALDI-TOF (Additional file 3: Fig. S3). It was observed that the molecular weights for all the fusion proteins were as expected. Biophysical characterization using a far UV CD spectrum of the purified fusion proteins post-solubilization was carried out (Fig. 7a, b). It indicated that the alpha-helicity increased twofold (from 29.2 to 54.1%) with the substitution of the valine at the 12^th^ position in *Cr*FAP65AH1; while there is only a ~ 15% increase (from 66.4 to 76.8%) in the alpha-helicity when the same is done with *Cr*FAP65AH2. The values for the alpha helicity were determined after processing the CD spectra with the BeStSel tool (Fig. 7c). It is known that proline residue causes a disruption in the protein's secondary structure and conforms to an alpha-helix or beta-sheet structure.

**Fig. 8:**
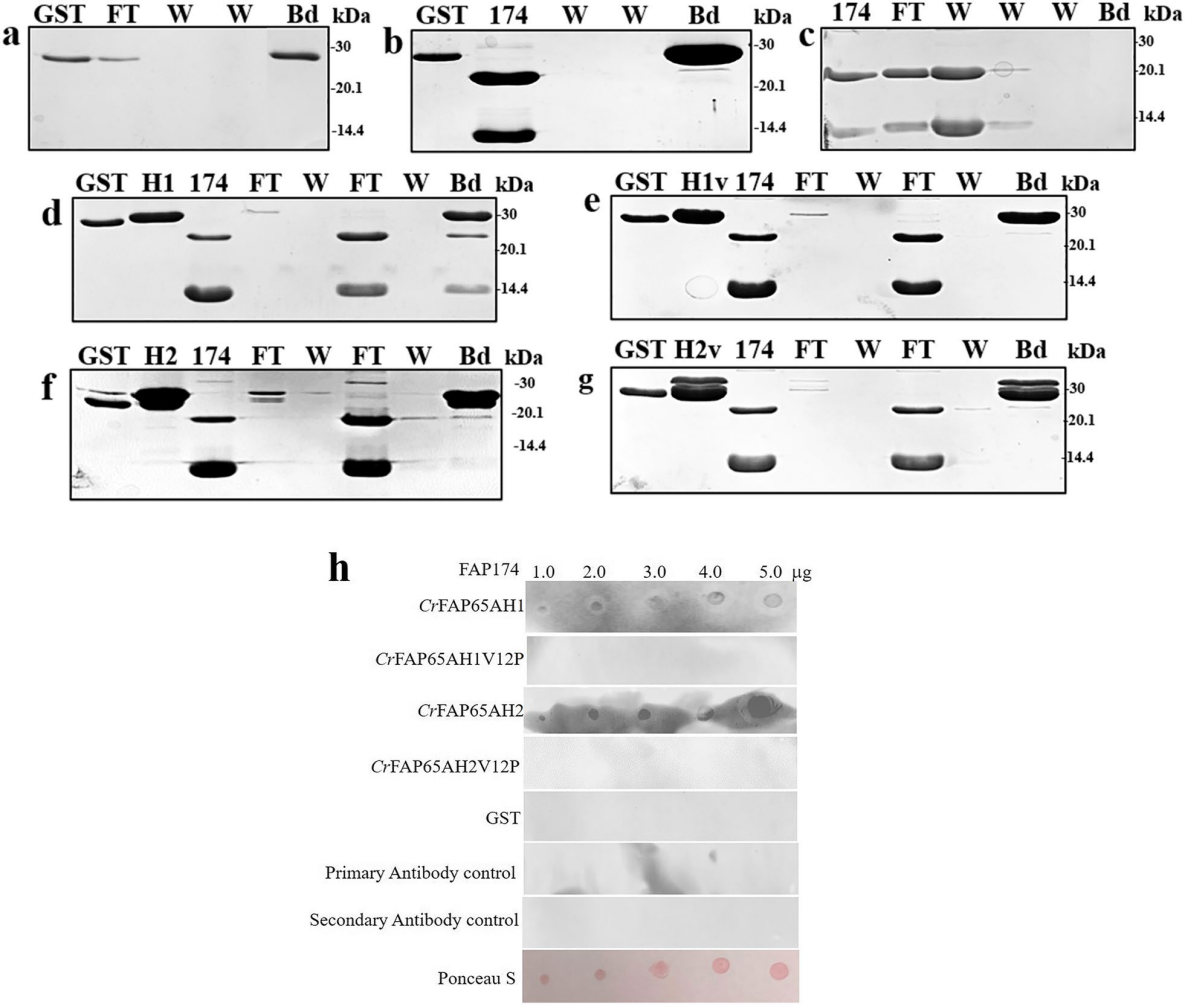
Pull-down and Dot-blot assay of the recombinant proteins with a known protein interactor, FAP174. **a** All four recombinant proteins purified to homogeneity were checked for their bioactivity by performing a pull-down assay with a known interactor. **a–c** Controls with GST alone binding to Glutathione-Sepharose beads, GST bound to the beads followed by addition of purified FAP174 (no binding seen when beads are electrophoresed on an SDS-PAGE gel, see lane marked Bd (for beads) and FAP174 bound to beads followed by FT (flow-through) and washes (W). Note the absence of FAP174 protein in **b** and **c**. **d**, **e** The *Cr*FAP65AH1 (H1 and its variant (H1v) pure proteins were individually used in the pull-down assay. FAP174 along with *Cr*FAP65AH1 (lane marked Bd in **d**) was seen, indicating a direct interaction. The variant, on the other hand, was not pulled down by FAP174 (see lane marked Bd in **e**). FAP174 was found to be present in the flow-through (lane marked FT in **e**). **f**, **g** The *Cr*FAP65AH2 (H2 and its variant (H2v) pure proteins were individually used in the pull-down assay. FAP174 along with a very low amount of *Cr*FAP65AH2 (lane marked Bd in **f**) was seen, indicating a direct, but weak interaction. The variant, on the other hand, was not pulled down by FAP174 (see lane marked Bd in **g**). Note the presence of FAP174 in the flow-through (lane marked FT in **g**). The brightness and contrast of all the images has been adjusted to 15%. **h** Dot blot followed by overlay assay indicated that *Cr*FAP65AH1 and *Cr*FAP65AH2 binds to FAP174 whereas no binding is observed in *Cr*FAP65AH1V12P, *Cr*FAP65AH2V12P, GST, Primary antibody control, Secondary antibody control

Taken together, our solubilization conditions with the use of mild denaturing reagents resulted in high-yield pure protein with the expected molecular weight and alpha-helical content. Further, the functionality of the purified recombinant proteins was ascertained using an interaction assay. Using highly purified FAP174 full-length protein as bait, a pull-down assay was performed individually with *Cr*FAP65AH1, *Cr*FAP65AH2 and their respective variants. These, which were bound to the Glutathione Sepharose beads worked as the prey. When the bands of these individual pull-downs were analysed on a denaturing gel, it was observed that *Cr*FAP65AH1 bound to both the monomer and dimer of FAP174 (Fig. 8d); the variant on the other hand did not show any interaction (Fig. 8e). When *Cr*FAP65AH2 was used, binding was evident but was weaker than that seen with *Cr*FAP65AH1 (Fig. 8f). Similarly, the variant of *Cr*FAP65AH2 did not show any interaction (Fig. 8g). The appropriate controls (only GST and GST with FAP174) showed no interaction with the respective recombinant proteins of *Cr*FAP65 (Fig. 8a–c). Dot blot was further performed to ascertain the binding between FAP174 and *Cr*FAP65AH1, *Cr*FAP65AH2 and their respective variants (Fig. 8h). It was observed that *Cr*FAP65AH1 and *Cr*FAP65AH2 bound to FAP174 whereas no binding was observed in the respective variants, GST, Primary and Secondary controls. We, therefore, surmise that the procedure used for purification yields bioactive recombinant proteins.

### Statistical analysis

We applied one-way ANOVA to determine whether there is a statistically significant difference between the protein yields within the eight purification techniques (independent variables). As the F statistic value for all was greater than the F critical value, we concluded that the test is significant. As the p-value was less than 0.05 we rejected the null hypothesis which was there is no significant difference between the protein yields within the respective purification techniques and accepted the alternative hypothesis which concludes that the difference between the protein yields within the purification techniques was statistically significant. Further, Tuckey’s *post-hoc* test confirmed the level of significance (Figs. 3e, 4c, 5b, 6b).

## Discussion

### Formation of inclusion bodies of GST-tagged amphipathic helices and their proline variants and initial steps of solubilization

The current study is an offshoot of an ongoing research work wherein FAP174 was used as bait to identify a protein complex from the flagella of *C. reinhardtii*. One of the direct interactors of FAP174 is an AKAP that was later annotated as FAP65 [31, 32]. It was shown to harbour two amphipathic helices, a signature sequence present in all AKAPs. Present in several stably folded proteins, amphipathic helices are found across kingdoms (viruses, bacteria, and eukaryotes) with a unique role in membrane targeting via protein-lipid and protein–protein interaction [38]. The important cellular functions they participate in include sensing membrane curvature, formation of tubular or spherical membrane intermediates, protecting membranes and lipid droplets, promoting membrane anchorage, and mediating membrane fission or scaffolding signalling components [38–42]. The list of proteins (membrane-bound, ion channels, apolipoproteins, AKAPs and lung surfactant) that are known to harbour amphipathic helices is long, and based on a detailed analysis of their physicochemical properties are categorized as 7 distinct classes (A, H, L, G, K, C, and M) [43, 44]. *Cr*FAP65 has been identified as a ciliary protein with a likely role in motility. *Cr*FAP65 is an AKAP, and keeping in mind the hydrophobic nature of the sequences, (Fig. 1) we used in silico tools to study the GRAVY index, a parameter that projects the hydrophobicity of the amphipathic helix. It was seen that *Cr*FAP65AH1 and its variant showed 0.412 and 0.18 GRAVY index, respectively indicating the hydrophobic nature of the protein fragments; whereas *Cr*FAP65AH2 and its variant showed − 0.089 and − 0.242 with not-so-high hydrophobic nature (Fig. 1d). However, as these values of *Cr*FAP65AH2 and *Cr*FAP65AH2V12P are closer towards zero, they may tend to display hydrophobic characteristics. Also, the amphipathic content was evident with the helical wheel projected using Heliquest and the hydrophobic moment indicated high hydrophobicity for *Cr*FAP65AH1 and its variant as compared to *Cr*FAP65AH2 and its variant (Fig. 1d). This feature has been observed for all amphipathic helices so far reported. Based on all these features, we have placed *Cr*FAP65AH1 and *Cr*FAP65AH2 into the globular (G) class [44]. There are different reports of amphipathic helices or amphipathic helix-containing proteins that form IBs upon over-expression in *E. coli*. These are also known to drive IB formation or simply form IBs in cells leading to diseases. For example, amphipathic polymers such as amphipols (Apols) help in stabilizing membrane proteins and are generally known to form IBs [45]. α-Synuclein, a 140 amino acid (a.a.) α-helix-rich protein contains an amphipathic helix and is known to form IBs in the cytoplasm. Such an aggregation of the protein is the main cause of Parkinson’s disease (PD) and Lewy body dementia (LBD) [46]. On the other hand, a self-assembling hydrophobic peptide GFIL8 (GFILGFIL) has been used to induce IB formation in *E. coli* [47]. However, the recombinant protein containing the coiled-coil domain does not need to maintain its helical structure in the IBs. Hence, we decided to fuse these amphipathic helices of FAP65 protein to a solubility-enhancer tag such as GST whose GRAVY index is -0.446, i.e. hydrophilic in nature [13]. Several studies have attempted the use of GST as a tag to produce recombinant amphipathic helices. Dengue virus non-structural protein 4A (NSP4A) whose 1–48 a.a. and its variant were cloned and overexpressed as GST-tagged fusion protein with yields of 4–5 mg/L of *E. coli* culture [48]. Yeast Bud3p involved in bud formation harbours an amphipathic helix and was GST-tagged for pull-down assays. However, no purification has been done with the Bud3p-GST protein [49]. Amphipathic helix-containing proteins belonging to the membrane binding BAR (Bin/Amphiphysin/Rvs) categories have also been produced with the GST tag, these being PICK1 (Protein Interacting with C Kinase), and ICA69 (Islet Cell Autoantigen 69 kDa). However, the buffers used were very different and the mention of IB is not evident [50]. Therefore, the amphipathic helices of *Cr*FAP65 (WT and the variants) were individually gene synthesized in pGEX-4T-1 and the choice of the host was *E. coli*, as it is with many investigators too. While GST-tagged fusion proteins rarely pose challenges in *E. coli*, the absence of a post-translational modification system and the formation of IBs are the two most unpopular aspects of this overexpression platform. To confirm whether these recombinant proteins form IBs we carried out a TEM analysis of *E. coli* cells overexpressing these amphipathic helices. It is known that the presence of hydrophobic patches in a recombinant protein causes it to aggregate due mainly to misfolding, thus leading to IB formation. These IBs generally appear as an electron-dense structure under the transmission electron microscope [28]. While in most cases, the ease of isolating the IB itself serves as one step of purification, the isolation of such aggregates might lead to bio-inactive recombinant protein. This might prove to be an undesirable bet. Therefore, we avoided the isolation of these IB aggregates that are already in their unfolded state. As expected, the overexpression in *E. coli* did not pose any challenges (Fig. 2a) with very high overexpression seen in samples induced for 6 h/37 °C. The induction was confirmed by determining the molecular weight of the protein and comparing it with the induced samples after electrophoresing them on an SDS-PAGE denaturing gel. While the over-expression for all four fusion proteins was abundant, the molecular weights were also as expected (Fig. 2a). We made use of a solubility enhancer tag (i.e. GST), low concentrations of the inducer (viz. IPTG), and a lysis and solubilization buffer that is regularly used for the solubilization of other recombinant flagellar proteins from *C. reinhardtii*. Despite these conditions, the fusion proteins were not completely soluble (Fig. 2b). By visual examination, it was quite evident that they all formed IBs (Fig. 2c), the least IB formation was seen with *Cr*FAP65AH2. We attribute the IB formation to several factors, such as the strong promoter (Tac) on the pGEX vector, probably the high copy number of the target genes and the hydrophobicity of the translated proteins [51]. Since these fusion proteins would eventually serve as baits in protein interaction assays, recovery of bioactive purified products by using milder treatments was sought. Hence, avoiding the use of harsh chaotropic or utilizing mild denaturing conditions for solubilization was the primary goal with the hope to obtain bioactive fusion protein. Studies on the use of non-denaturing agents such as *N*-lauryl sarcosine, dimethyl sulfoxide (DMSO), 5% *n*-propanol, mild non-ionic detergents, high pH buffers, and low denaturant concentration that preserve the native-like state of the fusion proteins have been reported [52–57]. To solubilize IBs a combination of denaturants such as Sodium do-decyl sulphate (SDS), urea and organic solvents such as 40% (*v/v*) 1-Propanol and 20% (*v/v*) 2-Butanol in an acidic pH (range of 2–3) are used [58]. Chaperones have the ability to transiently bind to the hydrophobic region of a protein and this binding avoids IB formation. Recently, the use of nanobodies as what is referred to as solubilization chaperones are preferred. These nanobodies can detect the discontinuous amino acids of a native protein structure thereby stabilizing it. Together, over-expressing an epitope EPEA tag (Glutamic acid-Proline-Glutamic acid-Alanine) bound to the recombinant protein and an anti-EPEA conjugated nanobody which is supposed to recognize each other thereby aiding in soluble protein production [59]. Our aim involved the use of a systematic design of experiments that tested the effect of salt, increasing buffer volume, adding a commercial solubilizing concoction (BugBuster^®^), using a mild detergent such as IGEPAL CA-630 and mechanical lysis using sonication. This design, singly or in combination, was aimed at gently disrupting the interactions in the IB aggregates that are ionic, hydrophobic, and have disulphide bridges or van der Waals forces.

When the induced cell pellets for each fusion protein were individually tested for solubilization in the LSB-1 containing 300 mM NaCl, solubilization was not complete and > 50–70% of the fusion proteins were still found to be associated with the pellets (Fig. 2b), indicating that the conditions in this buffer were unable to unfold the fusion proteins from the aggregates of IBs. Hence, the systematic strategy for solubilization that we adopted was to first target the salt present in the lysis and solubilization buffer and replace it with three different concentrations, viz. 75, 150 and 300 mM. Several inorganic and organic salts have in the past been used to denature or solubilize proteins from IBs. Inorganic salts are known to denature proteins when used at concentrations > 1 M. Of these, NaCl and KCl are the most popular as these are not only easily dialyzable but are successful in selectively extracting membrane proteins, as well [26]. NaCl at the concentration we used (300 mM) may contribute to a salting-out effect. It is known that lower concentrations of NaCl (< 200 mM) create a salting-in, an effect that is useful for solubilization. Hence, we used three concentrations of NaCl (75, 150 and 300 mM) and compared the solubilization in the presence and absence of a cell-lysing commercial reagent, viz. BugBuster^®^. It may be noted that the lysis of cells is one of the major contributory factors to the yield of purified proteins. The more the lysis, the more accessibility would be for the components in the buffer towards accessing the IBs and therefore solubilization. For effective solubilization, we used BugBuster^®^ which contains non-ionic and zwitterionic detergents (see user protocol TB245 Rev. F 1108, pages 1–7 of BugBuster^®^ Protein Extraction Reagent, Novagen). Next, we decided on the means for gauging or monitoring solubilization. Besides using denaturing gel electrophoresis, we also determined the total soluble protein content in the supernatants using Bradford’s reagent. The gels were used to semi-quantitate the band intensity using ImageJ. When such an experiment (with/without BugBuster^®^ and increasing NaCl) was performed, (Fig. 3a, b), the induced bands were quantitated for their respective intensities (Fig. 3d). The induced band intensity and the total soluble protein in the supernatants (Fig. 3c) were highest in the treatment that received BugBuster^®^ and 150 mM NaCl. Although there was no apparent difference in the three treatments, a one-way ANOVA with Tuckey analysis revealed that the total soluble protein in the supernatants obtained using 150 mM NaCl in LSB-2 was most significant over the other treatments (Fig. 3e). It was therefore decided to use this condition (lysis buffer containing 150 mM NaCl, i.e. LSB-2) for further solubilization. At this stage, two possibilities exist, either all the cells might not have been lysed with BugBuster^®^, or given the ionic strength of the buffer with 150 mM NaCl and the buffer volume (1250 μL), there already exists an equilibrium between the aggregated protein molecules in the IBs versus the folded protein molecules in the soluble fraction (supernatant). The latter is true when non-denaturing buffers are used for solubilizing IBs, especially without the use of any drastic or strong solubilization agent(s). This has been reported in a few cases, such as *N*-acetyl-d-glucosamine 2-epimerase IBs have been solubilized using Tris–HCl buffer, pH 7.0 [60], Granulocyte Colony-Stimulating Factor (G-CSF), His7dN6TNF-α (His-tagged, N-terminally truncated form of tumour necrosis factor) and GFP (green fluorescent protein) wherein a lower temperature (25^o^C) of induction was used [61]. It might also be possible that as in non-classical IBs, the unfolded aggregates might exist along with native-like structures, the latter being easily soluble. At this stage, we suspected that the amphipathic helix fusion proteins were partitioned between the insoluble aggregates and soluble fractions. Earlier studies have shown that protein solubility is also affected by other co-solvents that can bind to the protein or change the structure of water [62]. The best starting point was therefore to increase the lysis and solubilization buffer volume in the presence of BugBuster^®^. We, therefore, used 1875 and 3750 μL in the hope that detergents in the BugBuster^®^ would solubilize the fusion proteins from the IBs and the increased volume would in turn shift the equilibrium towards solubilization. Having tested this design, we found that the band intensity increased ~ 10–12% with every rise in the volume from 1250 through 1875 to 3750 μL (Fig. 4b). The total soluble protein content in the supernatants was also calculated (Fig. 4b), it was found that the highest protein content was seen when the LSB-2 volume was 1875 μL. On the other hand, the total soluble protein content in the supernatant dropped by ~ 50%, partly because of the dilution. However, the band intensity indicated a selective increase in the fusion protein. This incongruency in the band intensity and total soluble protein content in the supernatant prompted us to purify the protein to near homogeneity using affinity chromatography. The yield of the finally dialyzed pure protein was calculated as mg protein/L of *E. coli* culture. The yield for 3750 μL showed > two-fold increase over that of 1875 μL and > four-fold increase over that obtained from 1250 μL (Fig. 4b). Further, these values were found to be statistically significant when one-way ANOVA with Tuckey analysis was performed (Fig. 4c). However, this increase in the pure protein yield was not as high as one would anticipate for a GST-tagged fusion protein. This also tempted us to believe that the IBs are probably non-classical as they were solubilized in the presence of mild detergents and low concentrations of NaCl with a shift in the equilibrium of folded protein from the IBs upon dilution of the cell lysate. However, the question that remained unaddressed was that of the lysis of the *E. coli* cells. Hence, the next step was to try these designs either singly or in combination. Since purification of the protein from the supernatant was the confirmatory test of selective solubility of the fusion protein, subsequent tests used the supernatants for purification and thus calculation of the purified protein yields.

### Comparison of the final yields of the purified fusion proteins from all treatments for an accurate understanding of the optimum solubilization condition

Once assured that the fusion protein was soluble in the conditions mentioned earlier (3750 μL of LSB-3 with BugBuster^®^) albeit, with not very high yield, we wanted to ensure complete cell lysis using sonication. Knowing that these IBs behave like the non-classical ones, we also tested the effect of a mild non-ionic detergent, IGEPAL CA-630. Hence, in the subsequent exhaustive design, we used the following conditions for solubilization:

1. BugBuster^®^ (with and without sonication)
2. LSB-3 (with and without sonication)
3. LSB-3 incubated with BugBuster^®^ (with and without sonication)
4. LSB-3 incubated with BugBuster^®^ treated with IGEPAL (with and without sonication).

The supernatants from these treatments were individually subjected to fusion protein purification, and the purified dialyzed protein was used to estimate the total yield per litre of *E. coli* culture. The graph so obtained indicated that while solubilization with BugBuster^®^ increased the yield to ~ 6.5 mg/L of *E. coli* culture, the conditions wherein sonication was used increased the protein yield to > 7.5 mg/L of *E. coli* culture. In contrast to the lowest yield (~ 2 mg/L of *E. coli* culture), a tenfold yield (~ 20 mg/L of *E. coli* culture) was obtained with LSB-3 incubated with BugBuster^®^ (with sonication; Fig. 5a). We also observed that IGEPAL CA-630 decreased the yield which meant that solubilization or cell lysis was inhibited (Fig. 5a). IGEPAL CA-630, like all detergents, is being used as a surfactant which by virtue of its amphiphilic property aids the process of cell lysis by disrupting the cell membrane thereby releasing intracellular material. IGEPAL CA-630 (IUPAC name octylphenoxypolyethoxyethanol) (Sigma-Aldrich Catalogue no. 56741) is a non-ionic, non-denaturing mild detergent and is completely miscible in water. The hydrophobic-hydrophilic balance for the detergent is 13.1 with a crucial role for both octylphenol and ethylene oxide. This allows it to break protein-lipid and lipid-lipid interactions, but not protein–protein interactions. IGEPAL CA-630 has bulky non-polar heads that generally do not exhibit cooperative binding, much as seen with ionic detergents. Due to this property, it will efficiently disrupt membranes and not penetrate native structures. Our results indicate that IGEPAL CA-630 is certainly not a substitute for sonication; in fact, it acts as an inhibitor of solubilization. Due to its interference with protein estimation assays, we ensured that dialysis completely removed the detergent before we embarked on the protein estimation assays. Hence, the values calculated for the pure protein yields are authentic and significant as analyzed using one-way ANOVA with Tuckey (Fig. 5b).

When we compared the yields of GST-tagged fusion proteins as reported in the literature with the current study, we found few reports which suggested that GST is a poor solubility tag as compared to commonly used fusion tags as their protein yields were very low (Efna1 − 0.06 mg/L, CDK2 − 2.17 mg/L) [63]. Another GST-tagged amphipathic helix from the N-terminal of the dengue virus non-structural protein 4A (NSP-4A) produced low yields (4–5 mg/L of *E. coli* culture [48]. In yet another report, > 27 genes (without any amphipathic helices) were cloned with the GST tag. Of these, only seven produced 50% solubility (SMPX_HUMAN, HBP1_HUMAN, IPKA_HUMAN), the others were either present in very low amounts (MAR1_HUMAN, APR_HUMAN) or were not solubilized at all (STP1_HUMAN, BTG1_HUMAN, MGN_HUMAN) [64, 65]. An immunomodulatory protein from *Ganoderma tsugae* containing an N-terminal amphipathic helix when over-expressed in *E. coli* as a GST-tagged fusion protein gives rise to 20 mg/L of *E. coli* culture [66].

### Quality checks and structural analysis of fusion proteins by Circular Dichroism

Since the pure protein yield was maximum with LSB-3 incubated with BugBuster^®^ followed by sonication, this treatment was applied with *Cr*FAP65AH2 and the variants (*Cr*FAP65AH1V12P and *Cr*FAP65AH2V12P). The yield for *Cr*FAP65AH1 was lower than that obtained for *Cr*FAP65AH1V12P, and the reason for this is not known (Fig. 6a). It may be that the substitution of proline introduces more helicity in the sequence. On the other hand, *Cr*FAP65AH2 and its variant, *Cr*FAP65AH2V12P exhibited a very high yield (30–45 mg/L of *E. coli* culture; Fig. 6a). However, upon SDS-PAGE analysis and silver staining, it was observed that the *Cr*FAP65AH2 (WT and variant) was susceptible to degradation, and we attribute this high yield to the intact and degraded protein. It may be added that this degradation was seen even in the induced pellets and has no correlation to the solubilization process (data not shown), and the degraded 26 kDa protein is GST (Fig. 6A, inset). Taken together, the average yield for all the fusion proteins could be estimated as ~ 20 mg/L of *E. coli* culture. Such degradation has been observed in GST-tagged fusion proteins generated for truncated versions of a small G-protein (ArfGAP1) that harbours amphipathic helices. These seemed quite soluble in the extracts but showed degradation of the GST [67]. The authors attributed this to the fusion protein being soluble in the *E. coli* cells and being simultaneously vulnerable to proteases. It is also reported that the GST tag co-purifies itself along with the recombinant fusion protein as translation stops prematurely [68]. Given that *Cr*FAP65AH2 and its variant are less hydrophobic, we too extend this reasoning. Additionally, the use of IGEPAL-CA630 in the buffer inhibited the protein yield. This was ascertained statistically using one-way ANOVA with Tuckey analysis (Fig. 6b).

Since we expect the native-like structures of the fusion proteins to be preserved in the solubilization experiments that we tested, which also bypassed the refolding step (and, saved time), we wanted to ascertain the intactness of the secondary structure. Amphipathic helices have both hydrophobic and hydrophilic a.a. residues arranged in such a manner that the helix so formed creates two faces, one being hydrophobic and the other hydrophilic facing the opposite side. Such sequences have an inherent property of folding into helical structures upon contact with polar/non-polar interfaces. Hence, we estimated the alpha-helical content of amphipathic helices of *Cr*FAP65 using circular dichroism and the BestSel tool (Fig. 7a–c). The alpha-helical content was 29% and 66% respectively for *Cr*FAP65AH1 and *Cr*FAP65AH2 which increased with the substitution of the valine at 2020 and 2231 positions, respectively with proline from 29% for *Cr*FAP65AH1 to 54% for the variant (*Cr*FAP65AH1V12P). Again, the same was observed for the alpha-helical content for *Cr*FAP65AH2 that increased from 66 to 77% for the variant (*Cr*FAP65AH2V12P) (Fig. 7c). This was the first time that we estimated the alpha-helical content of the amphipathic helices. We further compared these values with those reported in the literature. For example, when a model amphipathic peptide (Ac-Gly-Ala-Glu-Lys-Ala-Ala-Lys-Glu-Ala-Glu-Lys-Ala-Ala-Lys-Glu-Ala- Glu-Lys-amide) was designed, and its alpha-helical content was measured using CD and 2D-NMR spectroscopies, it was shown to contain 65% alpha-helical structure [69]. The N-terminus Myristoylated-ADP Ribosylation Factor 1 (Myr-ADPR1) peptide has been shown to adopt a nearly 100% α-helical structure as determined by CD [70]. The human apolipoprotein C-1 has an amphipathic helix with alpha-helical content that increases to 65–75% when bound to phospholipids [71]. Certain GST-tagged amphipathic helices of ArfGAP1 (a small G-protein) are quite unstructured in solution, however, upon binding to liposomes, they do exhibit 25–48% alpha-helical content. ArfGAP1 responds to membrane curvature through the folding of a lipid packing sensor motif [70]. As for the increase in the alpha-helical content with substitution of value with proline, we note that despite proline being accepted as the helical breaker in an aqueous medium, reports of its function otherwise have been observed when they are in membrane environments than in water. The presence of proline in long alpha helices also helps in the proper folding of the proteins [72]. Proline has also been observed to increase the thermal stability of the protein as well as the alpha-helical conformation of the protein at high temperatures in presence of 2-propanol [73].

Since the secondary structure of the recombinant proteins was found intact, the functionality was further verified using a pull-down assay. FAP174 is an established interactor of AKAP240 i.e. *Cr*FAP65 [31, 32]. Hence, the full-length purified 6XHis-tagged recombinant protein purified using Ni–NTA affinity chromatography was used as prey for the interaction with *Cr*FAP65AH1, *Cr*FAP65AH2 and their respective variants (Fig. 8a–g). Using appropriate controls (Fig. 8a–c), it was observed that *Cr*FAP65AH1 bound strongly with FAP174, while *Cr*FAP65AH2 bound weakly (Fig. 8d, f). As expected, the proline variants did not exhibit any binding with FAP174 (Fig. 8e, g). In order to further confirm the bioactivity of these recombinant protein, dot blot was also carried out wherein increasing concentration of FAP174 on the blot was overlayed with CrFAP65AH1, *Cr*FAP65AH2 and their respective variants. The results obtained were like the pull-down assay wherein the *Cr*FAP65AH1 strongly bound to FAP174 as compared to *Cr*FAP65-AH2. However, no binding was seen in the variants as expected. These results indicate that the procedure used to purify *Cr*FAP65 amphipathic helices and their variants yields functional recombinant proteins.

## Conclusion

**Fig. 9:**
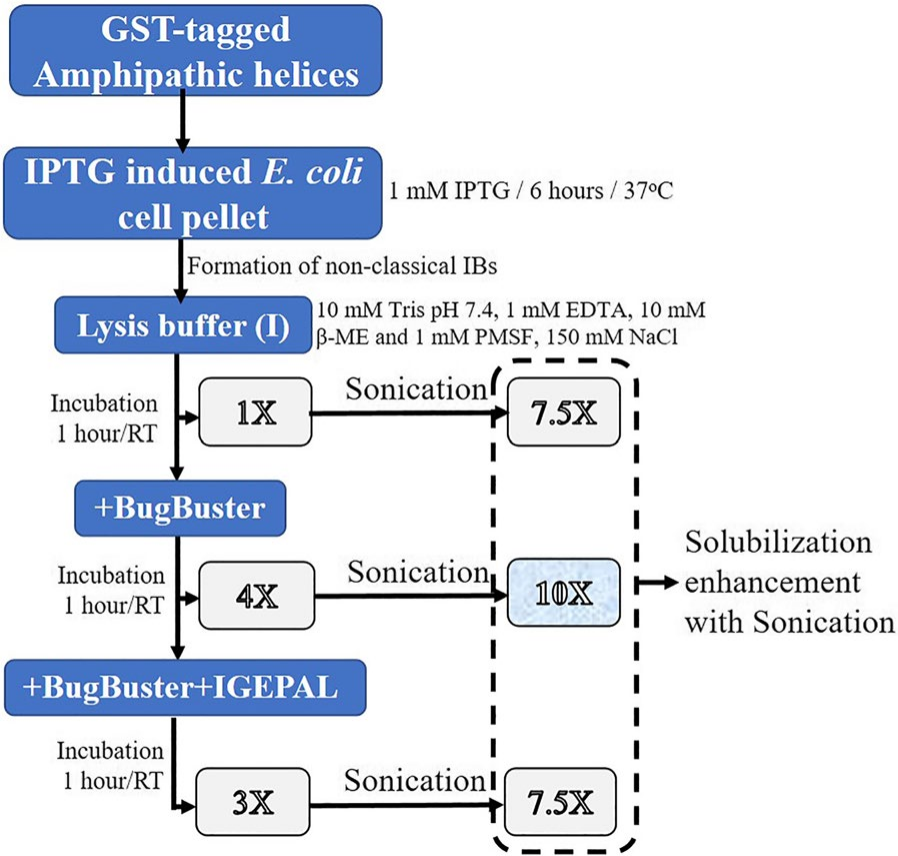
Workflow for the solubilization of GST-tagged amphipathic helices that form IBs. Note the increase in fold yield upon sonication and the decrease with IGEPAL CA-360

The current study enumerates a step-by-step design of experiments for the solubilization of IB proteins (probably of the non-classical type) for two amphipathic helices harboured on an AKAP (FAP65) that were surprisingly insoluble even after fusion with GST, a commonly used solubility-enhancer tag while producing recombinant proteins. We have successfully expressed the GST-tagged fusion proteins and using mild denaturing conditions have solubilized and purified the amphipathic helices in *Cr*FAP65. Although the tag is supposed to aid in solubilization, the proteins form IBs and it is the optimized lysis and solubilization buffer and mechanical breakage of cells that assist in the solubilization and further purification of the proteins. Amphipathic helices are a very important class of alpha helices present in membrane proteins, are useful antimicrobial and anticancer peptides, have an anti-inflammatory effect, are inhibitors of DNA and RNA viruses, are useful lipid droplet coaters, etc. Hence, its medical and pharmaceutical use relates to producing highly pure and bioactive molecules in this category. Given the amphipathic nature, its production can become very challenging. We, therefore, offer a mild denaturing procedure (Fig. 9) that not only helps bypass the re-folding step but also produces high-yield (~ 20 mg/L of *E. coli* culture) functional fusion proteins. If the GST tag is to be removed, a protease cleavage site may also be introduced if not present in the vector.

## Supplementary Information

**Additional file 1: Figure S1.** Plasmid vector map of pGEX-4T-1 and the position where the inserts were cloned.

**Additional file 2: Figure S2.** Transmission electron microscopy images of *E. coli* BL21 DE3 (a) Induced *Cr*FAP65AH1, (b) Induced *Cr*FAP65AH2, (c) Induced *Cr*FAP65AH1V12P and (d) Induced *Cr*FAP65AH2V12P.

**Additional file 3: Figure S3.** MALDI-TOF spectra for the affinity-purified and dialyzed recombinant proteins (a) *Cr*FAP65AH1, (b) *Cr*FAP65AH1V12P, (c) *Cr*FAP65AH2 and (d) *Cr*FAP65AH2V12P.

## Data Availability

All the data generated or analyzed during this study are currently not available because a relevant unsubmitted manuscript is in progress.
