# Supplementary figures and images for: Strategic optimization of conditions for the solubilization of GST-tagged amphipathic helix-containing ciliary proteins overexpressed as inclusion bodies in E. coli
Source: Microb Cell Fact. 2022 Dec 12;21:258. doi: 10.1186/s12934-022-01979-y (PMC9746132; doi:10.1186/s12934-022-01979-y)

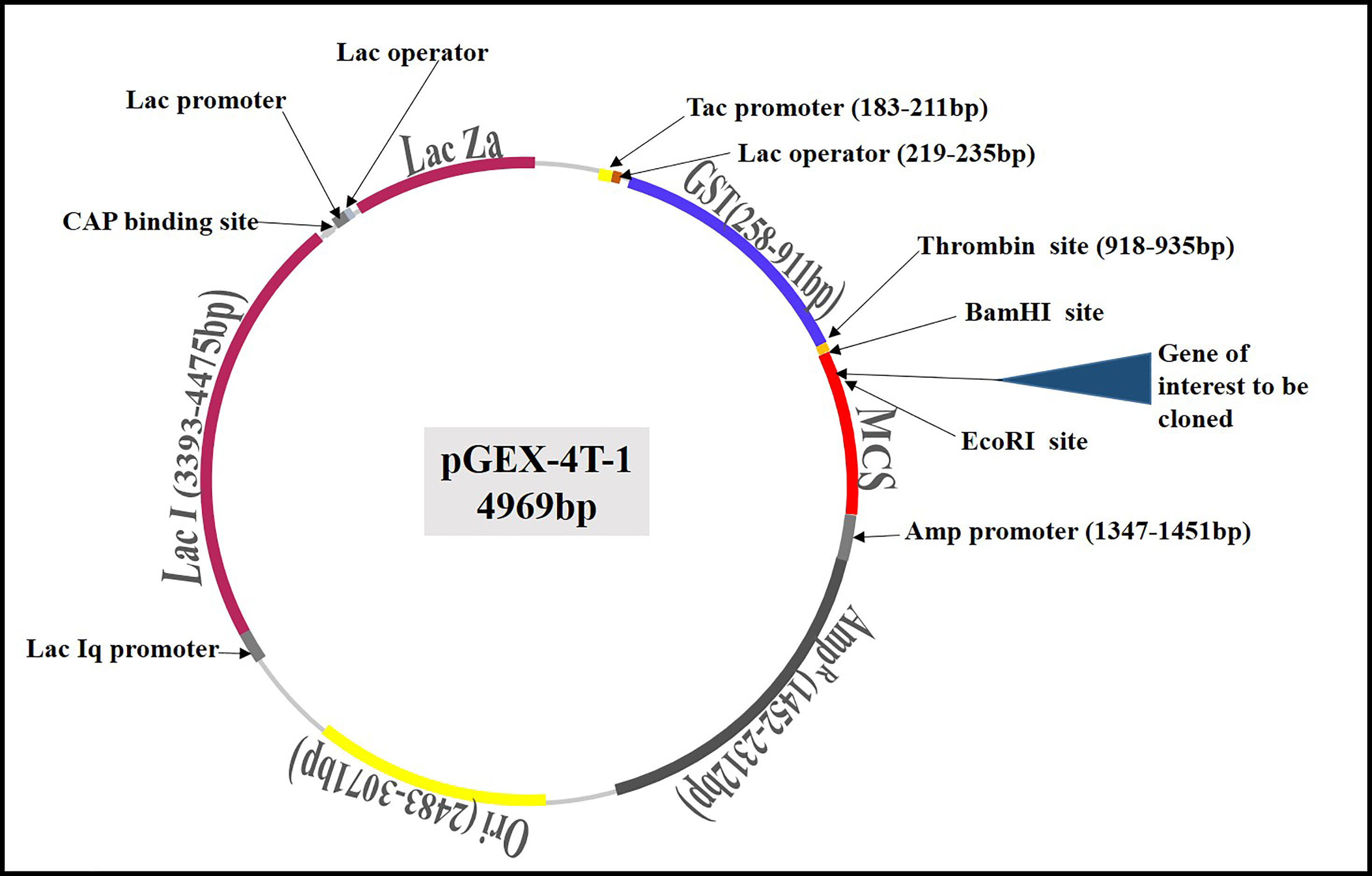

Supplement: Supplementary file 1 — Additional file 1: Figure S1. Plasmid vector map of pGEX-4T-1 and the position where the inserts were cloned. [file 12934_2022_1979_MOESM1_ESM.jpg]

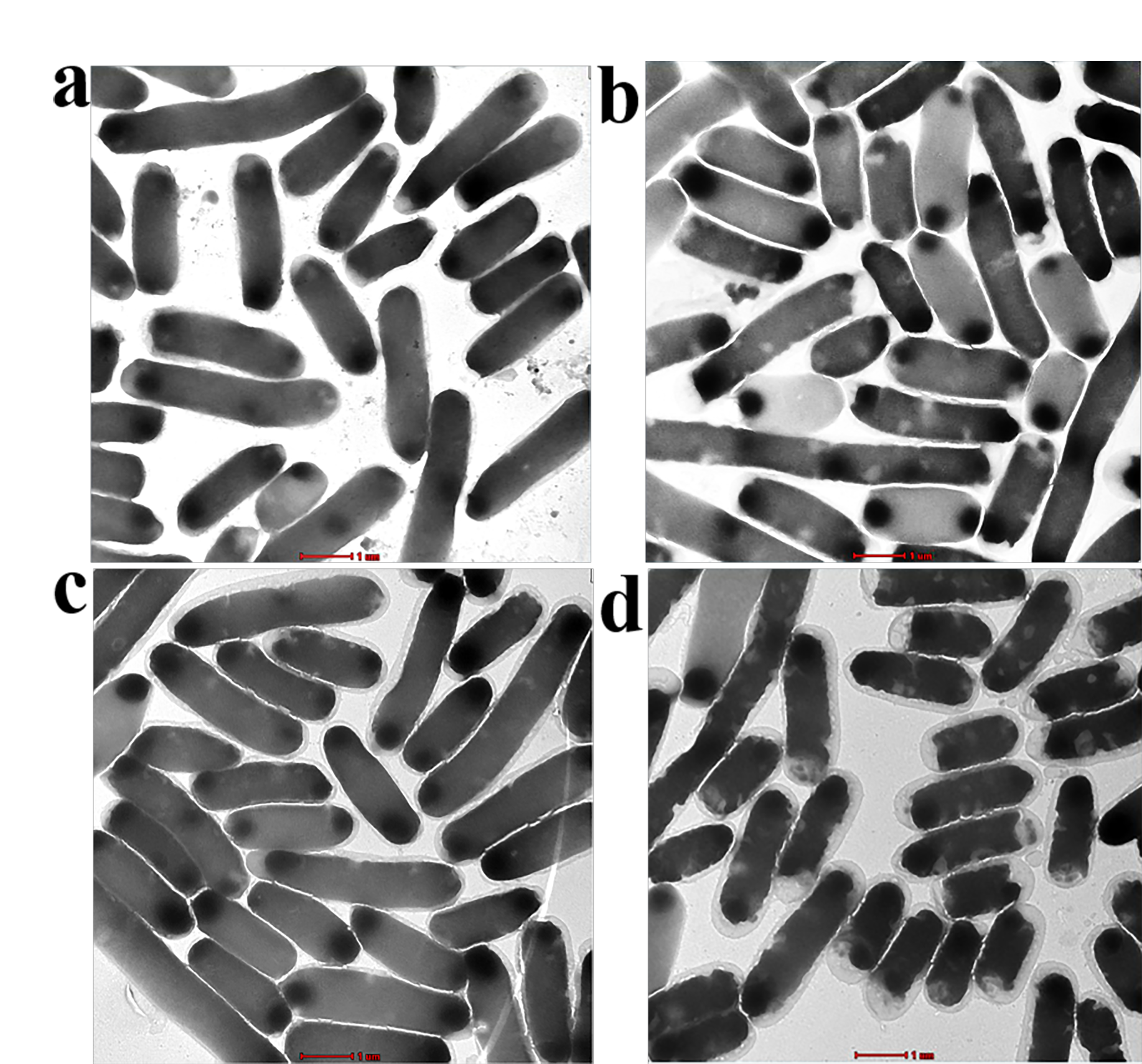

Supplement: Supplementary file 2 — Additional file 2: Figure S2. Transmission electron microscopy images of E. coli BL21 DE3 (a) Induced CrFAP65AH1, (b) Induced CrFAP65AH2, (c) Induced CrFAP65AH1V12P and (d) Induced CrFAP65AH2V12P. [file 12934_2022_1979_MOESM2_ESM.tif]

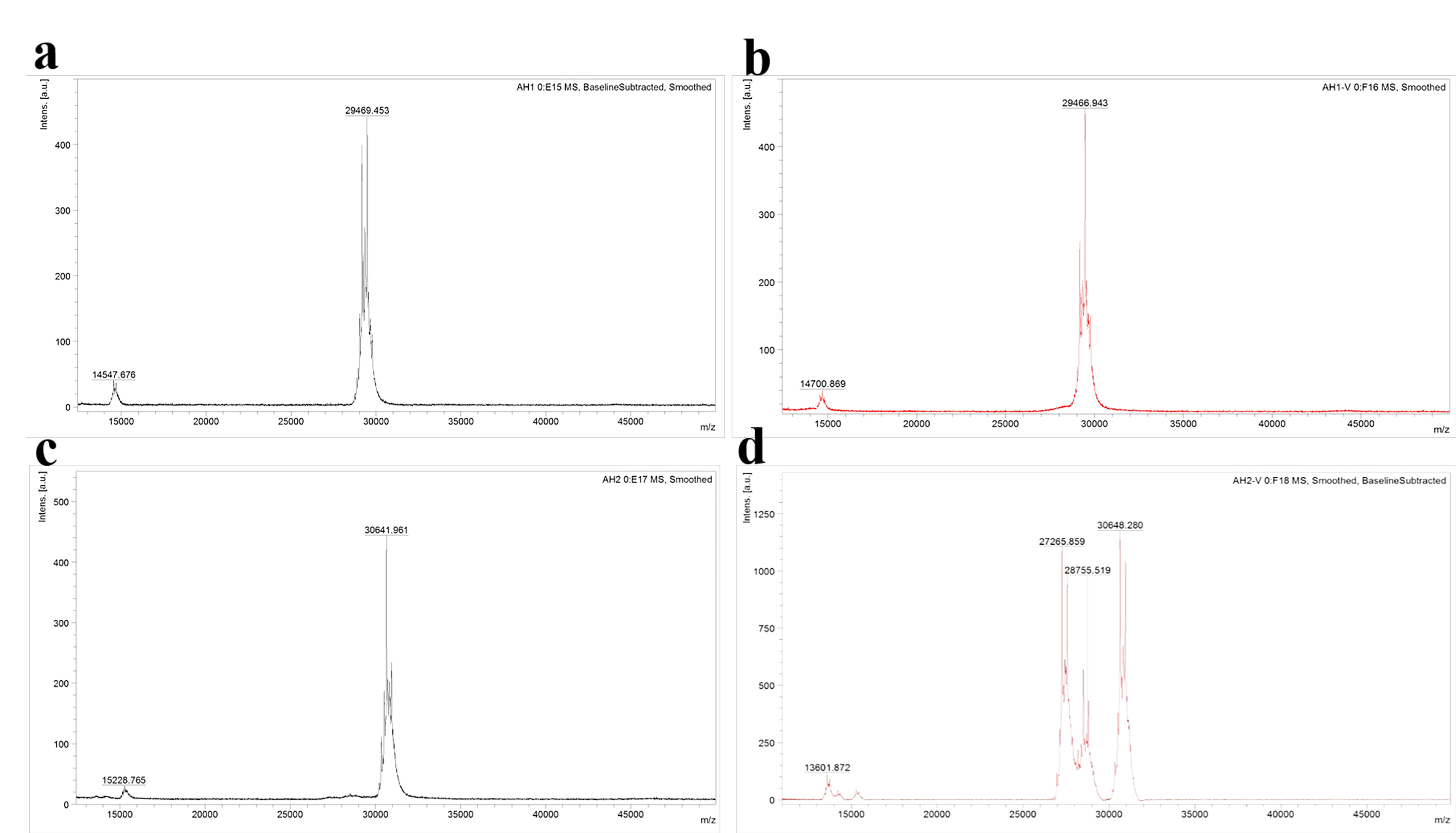

Supplement: Supplementary file 3 — Additional file 3: Figure S3. MALDI-TOF spectra for the affinity-purified and dialyzed recombinant proteins (a) CrFAP65AH1, (b) CrFAP65AH1V12P, (c) CrFAP65AH2 and (d) CrFAP65AH2V12P. [file 12934_2022_1979_MOESM3_ESM.tif]
